# In Vivo Clonal Analysis Reveals Development Heterogeneity of Oligodendrocyte Precursor Cells Derived from Distinct Germinal Zones

**DOI:** 10.1002/advs.202102274

**Published:** 2021-08-16

**Authors:** Rui Liu, Yinhang Jia, Peng Guo, Wenhong Jiang, Ruiliang Bai, Chong Liu

**Affiliations:** ^1^ Department of Neurobiology and Department of Neurosurgery of Second Affiliated Hospital, Zhejiang University School of Medicine Hangzhou Zhejiang 310058 P.R. China; ^2^ Department of Pathology and Pathophysiology Zhejiang University School of Medicine Hangzhou Zhejiang 310058 P.R. China; ^3^ NHC and CAMS Key Laboratory of Medical Neurobiology MOE Frontier Science Center for Brain Science and Brain‐machine Integration School of Brain Science and Brain Medicine Zhejiang University Hangzhou Zhejiang 310058 P.R. China; ^4^ School of Medicine Zhejiang University City College Hangzhou Zhejiang 310015 P.R. China; ^5^ Department of Physical Medicine and Rehabilitation of The Affiliated Sir Run Shumen Shaw Hospital Interdisciplinary Institute of Neuroscience and Technology Zhejiang University School of Medicine Hangzhou Zhejiang 310029 P.R. China

**Keywords:** developmental origin, heterogeneity, homeostasis, in vivo clonal analysis, *NF1*, oligodendrocyte precursor cell

## Abstract

Mounting evidence supports that oligodendrocyte precursor cells (OPCs) play important roles in maintaining the integrity of normal brains, and that their dysfunction is the etiology of numerous severe neurological diseases. OPCs exhibit diverse heterogeneity in the adult brain, and distinct germinal zones of the embryonic brain contribute to OPC genesis. However, it remains obscure whether developmental origins shape OPC heterogeneity in the adult brain. Here, an in vivo clonal analysis approach is developed to address this. By combining OPC‐specific transgenes, in utero electroporation, and the PiggyBac transposon system, the lineages of individual neonatal OPCs derived from either dorsal or ventral embryonic germinal zones are traced, and the landscape of their trajectories is comprehensively described throughout development. Surprisingly, despite behaving indistinguishably in the brain before weaning, dorsally derived OPCs continuously expand throughout life, but ventrally derived OPCs eventually diminish. Importantly, clonal analysis supports the existence of an intrinsic cellular “clock” to control OPC expansion. Moreover, knockout of *NF1* could circumvent the distinction of ventrally derived OPCs in the adult brain. Together, this work shows the importance of in vivo clonal analysis in studying stem/progenitor cell heterogeneity, and reveals that developmental origins play a role in determining OPC fate.

## Graphical abstract

A PiggyBac‐transposon‐based in vivo clonal analysis system is developed to delineate the trajectory landscape of individual oligodendrocyte precursor cells (OPCs). The authors show that early developmental origins determine OPC heterogeneity. Both cell fate and the division pattern of OPCs can be affected by their developmental origins. This study highlights the importance of clonal analysis in deciphering cellular heterogeneity in vivo.

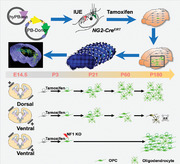

## 1 Introduction

Oligodendrocyte precursor cells (OPCs), also known as polydendrocytes, have been considered as the fourth major neural cell type in the central nervous system (CNS), in addition to neurons, astrocytes, and oligodendrocytes.^[^ ^1^ ^]^ It was previously believed that OPCs solely function as precursors during early development to generate mature oligodendrocytes, which produce myelin sheath to speed nerve impulses. Nevertheless, it was recently found that, even in the adult brain, OPCs constitute 4–8% of all cells and remain largely undifferentiated. And due to their capacity to proliferate throughout the life, OPCs are viewed as a major proliferation pool in the brain.^[^ ^2^, ^3^ ^]^

Mounting evidence supports that OPCs may function in complicated roles beyond providing mature oligodendrocytes, including the multipotentiality to transdifferentiate into neurons or astrocytes,^[^ ^4^ ^]^ interaction with neurons to form synapsis‐like structures,^[^ ^5^, ^6^ ^]^ and shaping the formation of neuronal circuits involved in motor learning.^[^ ^7^, ^8^ ^]^ Not surprisingly, dysfunction of OPCs has been associated with many serious CNS diseases, including multiple sclerosis,^[^ ^9^ ^]^ autism,^[^ ^10^, ^11^ ^]^ and schizophrenia.^[^ ^12^, ^13^ ^]^ In particular, owing to their strong potential to proliferate throughout life, OPCs have been proven to be important cellular origins for multiple tumors in the CNS, including adult glioblastoma, pediatric glioma, and medulloblastoma.^[^ ^14^, ^15^, ^16^, ^17^ ^]^ Therefore, delineating how OPCs develop and maintain their homeostatic status will provide insight into the function of these intriguing glial cells in both normal physiology and disease.

One fundamental question in OPC biology is whether OPCs in the CNS are intrinsically heterogenous and, if so, whether such heterogeneity is related to their early developmental origins.^[^ ^18^, ^19^ ^]^ Many studies appear to support that OPCs are heterogeneous. For instance, the morphology, cellular density, proliferation rate, differentiation potential, sensitivity to growth factors, electrophysiological property, and transcriptomes, have been reported variable for OPCs from distinct brain structures.^[^ ^2^, ^20^, ^21^, ^22^, ^23^, ^24^ ^]^ However, the extent to which such variation in cellular behaviors is attributed to intrinsic properties or solely regulated by their local environments remains elusive. Intriguingly, recent single‐cell RNA sequencing studies suggest that, at the transcriptional level, fetal, and postnatal OPCs from mouse and human brains exhibit similar transcriptional profiles, arguing for the homogenous nature of OPCs regardless of their locations^[^ ^8^, ^25^ ^]^—at least at the embryonic and early neonatal stages.

**Figure 1:**
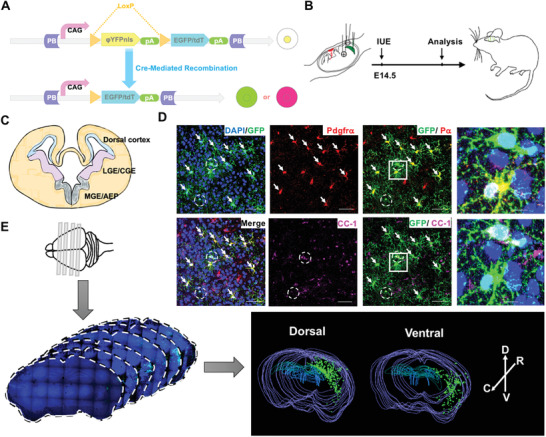
Development of PiggyBac transposon‐based in vivo lineage tracing system with single cell resolution. A) Configuration of the PiggyBac transposon reporter system designed in this study. By default, the cells integrated with the PB vector will express a nucleus‐localized yellow fluorescent protein (ΦYFP). The expression of cell‐type specific recombinase removes the ΦYFP coding sequence along with the stop signals (LoxP‐Stop‐LoxP,) and results the expression of EGFP/tdTomato. B) Diagram showing the procedure used to generate labeled OPCs by IUE. C) Diagram showing the anatomical structure of an embryonic mouse brain, wherein the three germinal zones functioning as the developmental origins of OPCs are labeled. D) Representative immunofluorescent staining images of brain sections from *NG2‐Cre* mouse brains electroporated with the PB: LoxP‐Stop‐LoxP (LSL)‐EGFP reporter. Arrows indicate the cells expressing Pdgfr*α* (the OPC marker). Dotted circles mark the cells expressing CC1 (the differentiated oligodendrocyte marker). P*α*, Pdgfr*α*. Scale bars, 50 µm. The right panels are the magnifications. Scale bars, 20 µm. E) 3D reconstruction from consecutive brain sections demonstrates the different distribution among OPC lineage cells with distinct origins analyzed at P21. Contours of the boundaries of brain sections, namely: the CC and the lateral ventricle, are marked by colored lines. Green dots represent the labeled oligodendrocyte lineage cells. D, dorsal. V, ventral. R, rostral. C, caudal.

The development of OPCs is complicated and highly dynamic. OPCs in the CNS have been thought to be derived from distinct germinal zones around the ventricle walls of the neural tube, with their generation under precise temporospatial control.^[^ ^26^, ^27^, ^28^, ^29^, ^30^ ^]^ An elegant genetic study by Richardson and his colleagues suggests that OPCs in the telencephalon are generated from distinct ventral and dorsal germinal zones, followed by three waves.^[^ ^31^ ^]^ On embryonic day (E)11.5, the first wave of OPCs is produced by Nkx2.1‐expressing progenitor cells in the medial ganglionic eminence (MGE) and the anterior entopeduncular area (AEP) on the ventral side of the brain. The subsequent wave of OPCs originates ventrally from the Gsh2 (Gsx2)‐positive precursors in the lateral and caudal ganglionic eminence (LGE and CGE) around E15.5. And the last wave of OPCs is from Emx1^+^ progenitor cells in the dorsal germinal zone after birth (see also **Figure** **1**C for the anatomical structures).

Intriguingly, lineage‐tracing experiments revealed that MGE‐APE‐derived OPCs are eventually eliminated during postnatal development, which suggests that OPCs from distinct developmental origins are intrinsically different and process distinct fates.^[^ ^31^ ^]^ Consistent with this notion, it was reported that ventrally derived OPCs require SHH signaling for their proper development, while dorsal OPCs are SHH‐independent.^[^ ^32^ ^]^ The complexity of developmental origins raises the question of whether the heterogeneity of OPCs in the adult brain is intrinsically imprinted by their origins or solely response to the local cues of the microenvironment. Intriguingly, ablation experiments showed that any one of the three sources of OPCs were genetically eliminated, while others would compensate for their loss in the brain, exhibiting the functional equivalence of OPCs regardless of their origins.^[^ ^31^ ^]^ Furthermore, single‐cell analysis suggested that ventrally and dorsally derived OPCs in the mouse brain were indistinguishable based solely on their transcriptomes, arguing that developmental origins play a role in determining the possible heterogeneity of OPCs.^[^ ^25^ ^]^ Therefore, despite the early pioneering studies clearly demonstrating the heterogeneity and complexity of OPCs, our understanding of the relationships between the origins and the heterogeneity of OPCs in the brain remains incomplete.

In this study, we developed an in vivo fate‐mapping system by combining transgenic mouse lines, orientation‐specified in utero electroporation (IUE), and the PiggyBac transposon‐based labeling system to perform bona fide in vivo clonal analysis, and traced the developmental trajectory of single neonatal OPCs originally derived from either the ventral or dorsal germinal zone. We found that developmental origins indeed play a role in determining the cellular fate of OPCs in the adult mouse brain. Although dorsally derived OPC clones continuously expanded, ventrally derived clones were eventually eliminated from the brain. However, the difference between dorsal and ventral clones was not prominent before the weaning age, therefore reconciling the conflicting results of previous lineage‐tracing and single‐cell sequencing studies. We also revealed that the patterns of early clonal expansion were different among OPCs derived from distinct germinal zones, and each exhibited a unique unitary production of progeny in a “quantum” way, suggesting the existence of an intrinsic cellular clock for OPCs in vivo. Importantly, we revealed that *NF1* deficiency can reprogram the fate of ventrally derived OPCs and circumvent their death in the adult brain.

## 2 Results

### 2.1 Development of a PiggyBac (PB) Transposon‐Based In Vivo Genetic System for Cell Type‐Specific Fate‐Mapping with Single‐Cell Resolution

To precisely map the fate of OPCs derived from distinct germinal zones with single‐cell resolution, we first developed an in vivo system by combining OPC‐specific Cre or Cre^ERT^ transgenes with a Cre‐dependent, PiggyBac (PB)‐based reporter system (Figure 1A,B). After co‐injection into the lateral ventricle with a non‐integrative plasmid that expressed a hyperactive form of PBase (HyperPBase), the PB reporter vector was delivered and stably integrated into the genome of the cells lining the lateral ventricle through IUE. Most of the cells lining the lateral ventricle are radial glia, which are the embryonic form of neural stem cells (NSC).^[^ ^33^ ^]^ Targeting the cells in the dorsal or ventral germinal zone was achieved by adjusting the orientation of the electrodes during IUE.

In the design of the final PB reporter vector, a LoxP‐Stop‐LoxP (LSL)‐EGFP/tdTomato cassette was positioned downstream of a strong and constitutive CAG promoter to label the desired cell type only upon Cre‐mediated recombination (Figure 1A). To visualize the cells successfully transfected with the PB vector, an open reading frame (ORF) encoding a nucleus‐localized yellow fluorescent protein (nΦYFP) was positioned between the two LoxP sites. Following the design, in the absence of Cre recombinase, the targeted cells express nΦYFP by default. Instead, if the cells express Cre recombinase, the nΦYFP cassette is removed, resulting in the expression of the downstream EGFP/tdTomato reporter. To ensure the strong and stable expression of the reporter, which is otherwise essential for lineage tracing studies, we further positioned two tandem insulator sequences on each side of the PB cassette to minimize potential silencing of the reporter gene after being integrated into the mouse genome.

To validate the feasibility of this system, we first evaluated the stable integration of the PB vector into the NSC genome by comparing the labeling pattern with and without hyperPBase (Figure S1A, Supporting Information). In the absence of hyperPBase, very few labeled cells could be found next to the SVZ upon dissection despite numerous labeled cells being detected in the upper layer of the cortex. In stark contrast, in the presence of hyperPBase, labeled cells could be easily detected next to the SVZ. Together, these observations validated that hyperPBase is necessary for the stable integration of the PB vector into the genome of embryonic NSCs.

Next, we performed IUE of the tdTomato PB reporter vector in *hGFAP‐Cre* mice to validate cell‐type specific labeling of the system. hGFAP‐Cre is known to be strongly expressed in the radial glia of the embryonic brain.^[^ ^34^ ^]^ As shown in Figure S1B, Supporting Information, tdTomato‐labeled cells were clearly observed in the region next to the ventricle of the *hGFAP‐Cre* mouse brain. In contrast, only nΦYFP‐labeled cells were detected in the wild‐type brain. These data confirmed that cell labeling by our PB reporter system was fully dependent on Cre recombinase.

Furthermore, we validated electroporation efficiency via electroporation of a non‐integrated plasmid expressing EGFP into distinct zones around the lateral ventricle. The comparable number of GFP^+^ cells proved that the initial labeling efficiency between the dorsal and ventral orientations was consistent (Figure S1C,D, Supporting Information).

### 2.2 Lineage Tracing of OPCs from Dorsal and Ventral Developmental Origins

To trace OPCs derived from initially labeled radial glia, we selected *NG2‐Cre or NG2‐Cre^ERT^* transgenes, both of which have been widely used to label and manipulate OPCs in vivo.^[^ ^23^, ^35^ ^]^ We first mapped the fate of dorsal and ventral NSC‐derived OPCs and their progeny at the population level using *NG2‐Cre*. Given the absence of temporal resolution, *NG2‐Cre* labeled all OPCs differentiated from initially labeled neural stem cells between the time point of IUE and histological analysis. We chose embryonic day14.5 (E14.5) to perform IUE for two reasons: 1) E14.5 was proven within a suitable time window to perform IUE with low abortion risk; and 2) covered the events to generate all dorsally derived OPCs and those from the ventral LGE/CGE. It may miss the peak of MGE/AEP‐derived OPCs (which starts after E12.5). Nevertheless, based on the previous study, MGE/AEP‐derived OPCs do not contribute significantly to the pool of OPCs in the adult brain (Figure 1C).^[^ ^31^ ^]^

We first validated the labeling pattern in our *NG2‐Cre* IUE model. As shown in Figure 1D, most labeled cells were either PDGFR*α* ^+^ or CC1^+^, indicating that they were derived from the OPC lineage. Although *NG2‐Cre* was known to label pericytes,^[^ ^36^ ^]^ we did not observe any EGFP^+^ cells that co‐expressed with the pericyte marker PDGFR*β* (Figure S2A, Supporting Information). One reason for this could be that IUE preferentially targeted neuroepithelia, but not pericyte precursors in our model. We did not detect any EGFP‐labeled astrocytes either (Figure S2B, Supporting Information). Some neurons (likely projection neurons based on their morphology) were found among EGFP‐labeled cells, especially in dorsally electroporated brains (data not shown). However, these labeled neurons likely resulted from direct leakage of *NG2‐Cre* rather than trans‐differentiation from the initially labeled OPC, as shown below by the in vivo clonal analysis.

Next, we examined the labeling patterns of dorsally and ventrally electroporated mouse brains. In the brains of postnatal day 21 (P21) electroporated with either orientation, a significant number of EGFP‐labeled cells could be detected. Consecutive tissue sectioning and 3D reconstruction revealed that OPC‐lineage cells derived from the dorsal and ventral germinal zones tended to populate distinct anatomical brain structures at this stage (Figure 1E). In general, in the brains from the dorsal IUE model, EGFP‐labeled OPC‐lineage cells tended to be localized more dorsally and vice versa.

**Figure 2:**
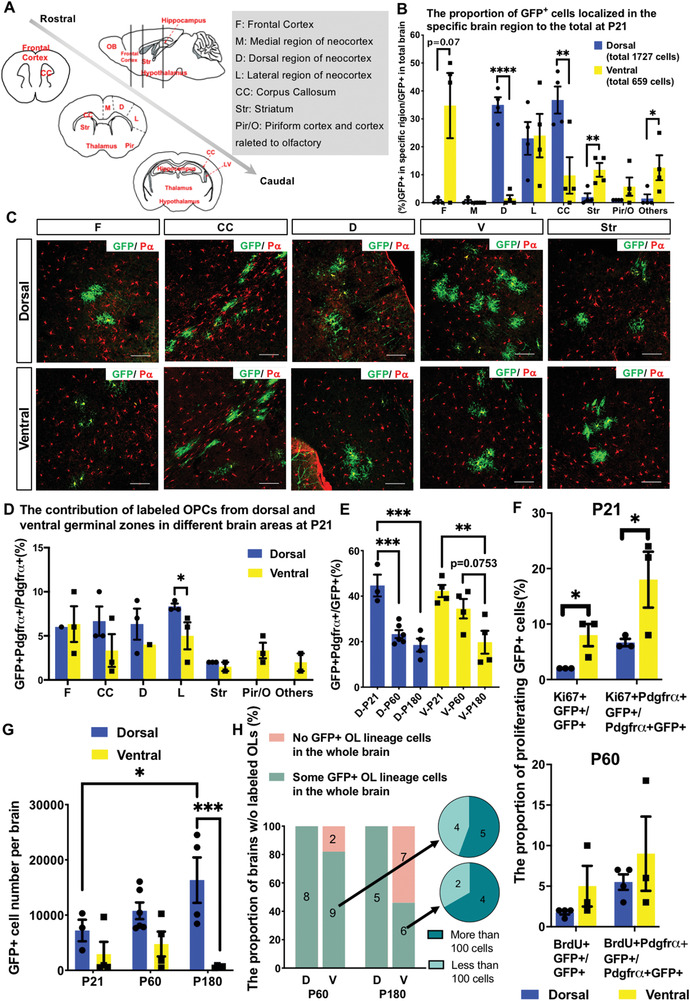
Lineage tracing at the populational level reveals that OPCs with different origins are heterogeneous. A) Diagram showing the anatomical sub‐structures of an adult mouse brain and their abbreviations where labeled OPCs populated. B) The distribution of labeled cells at P21 from the electroporation models as indicated. *N* = 4 mice for each group. Error bar: mean ± SEM. *N* = 4 mice for each group. Mann–Whitney test for comparison of group “F”, group “others”. T test for other groups. One‐tailed. *p < 0.05, **p < 0.01, ****p < 0.0001. C) Representative immunofluorescent staining images of the labeling pattern in the indicated brain areas. P*α*, Pdgfr*α*. Scale bars, 100 µm. D) The numbers of labeled OPC lineage cells from dorsal and ventral germinal zones in the indicated brain areas at P21. *N* = 4 mice for each group. Error bars: mean ± SEM. T test for group D, L, CC, and Str. Mann–Whitney test for other groups. One‐tailed. E) The differentiation potential of OPCs derived from dorsal or ventral origins. *N* = 4 mice for all ventral groups. For dorsal groups, *N* = 3 mice for P21, *N* = 7 for P60, and *N* = 4 for P180. Error bars: mean ± SEM. One‐way ANOVA, *p* = 0.0003 for D, *p* = 0.0113 for V. Tukey post‐hoc test, **p < 0.01, ***p < 0.001. F) The proliferation potential of labeled OPCs from dorsal and ventral germinal zones in different brain areas. Ki67 indicates the proliferating cells at P21. Treatment of BrdU for 8 days labeled the proliferating cells at P60. *N* = 3 mice for each group. Error bars: mean ± SEM. Mann–Whitney test for comparison of the proportion of BrdU positive green cells at P60. T test for others. One‐tailed. *p < 0.05. G) Total numbers of GFP positive cells in dorsally and ventrally electroporated brains harvested at indicated ages. Error bars: mean ± SEM. Two‐way ANOVA, *p* = 0.0556. Tukey post‐hoc test, *p < 0.05, ***p < 0.001. H) Number and proportion of available samples obtained from dorsal and ventral electroporation. The histogram shows how many brains contain cells or not. The pie chart shows how many cells labeled in a brain. The number of mice in each group is shown in the graph.

Using the mouse brain atlas as a reference (see also **Figure** **2**A),^[^ ^37^ ^]^ we confirmed that dorsally derived OPC‐lineage cells frequently populated the corpus callosum (CC), as well as the dorsal and lateral regions of the neocortex. In contrast, ventrally derived OPCs were more localized in structures such as the striatum, piriform cortex, and thalamus (Figure 2B, with their representative images shown in Figure 2C). We also noted that at this stage, labeled OPCs in the frontal cortex were mostly derived from the ventral origin, although this anatomical structure is in the dorsal part of the brain. Importantly, as shown in Figure 2D, although the total labeling efficiency of ventrally derived cells was slightly lower than that of dorsally derived cells, in some regions (such as the frontal cortex and the striatum), the absolute number of ventral derived OPCs was still higher than that from the dorsal ones. Therefore, the difference in distribution between dorsal and ventral derived OPCs is more likely associated with the property of OPCs per se, rather than the labeling efficiency. Our data also revealed that both dorsally and ventrally derived OPCs contributed to OPC‐lineage cells in the lateral region of the neocortex, although we cannot fully exclude that this overlapping pattern might have resulted from targeting the same cell population in different IUE models.

We then examined the differentiation potential of dorsal and ventral germinal zone‐derived OPCs by quantifying the ratio of OPCs and differentiated oligodendrocytes at three time points (P21, P60, and P180, respectively). We used the expression of PDGFR*α* as a criterion to distinguish OPCs from oligodendrocytes. As shown in Figure 2E, although among both germinal zone‐derived OPC‐lineage cells, the percentage of OPCs gradually decreased as the mice aged, and the OPC percentage from the dorsal origin exhibited a faster drop off compared to ventrally derived ones. This observation suggests that dorsal geminal zone‐derived OPCs possess a stronger potential to differentiate into mature oligodendrocytes than their ventrally derived counterparts.

Intriguingly, we consistently observed much a lower number of labeled cells in the ventral IUE model than in the dorsal model in aged brains. In fact, <100 or even no labeled OPC‐lineage cells were frequently detected in the entire brain from the ventral IUE model, although thousands of labeled cells could be readily detected in the dorsal IUE model (Figure 2G,H). The inefficiency in labeling of OPC‐lineage cells was unlikely due to the failure of IUEs, as EGFP^+^ neurons were easily detected in the same brain (data not shown). Thus, these data, together with the data from the clonal analysis, strongly suggest that OPC‐lineage cells originating from the ventral germinal zone tend to die out in the adult brain. Intriguingly, although ventrally derived OPCs/OLs eventually disappeared, the proliferation rate of ventrally derived OPCs was higher than that of their dorsal counterparts at an earlier stage (Figure 2F, with their representative images shown in Figure S3, Supporting Information). This may reflect the effort of ventrally derived OPCs to maximally maintain homeostasis to counteract the higher rate of cell death.

### 2.3 Optimization of the Electroporation Model for the In Vivo Clonal Analysis of OPCs

While the analysis at the population level suggests that dorsally and ventrally derived OPCs exhibited distinct behaviors on average, it cannot clearly delineate the developmental trajectory of individual OPCs. To address this question, we leveraged the technique of in vivo clonal analysis of single progenitors. Ideally, in vivo clonal analysis requires the labeling density in the organ/tissue of interest to be extremely sparse to be able to separate each initially labeled progenitor cell well. Thus, all progeny derived from the same progenitor cell are expected to cluster together and be physically isolated from other unrelated progenitor cells. This method assumes that the progeny of each clone does not migrate too far away from their original place, such as OPCs, hippocampal stem cells, and cortical neural progenitors.^[^ ^23^, ^38^, ^39^, ^40^ ^]^ For the clonal analysis with temporal resolution, it is also mandatory that the labeling exhibits no background, and that the labeling of cells is stringently controlled; otherwise, the start point of a clone examined would be inconclusive.

**Figure 3:**
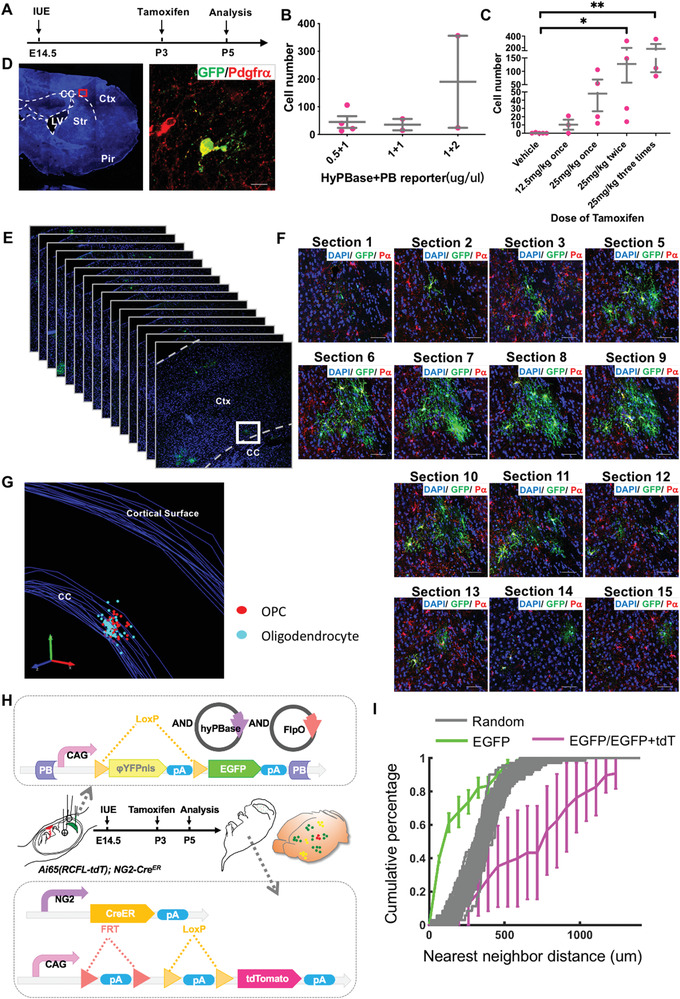
Development of the system for in vivo clonal analysis of OPCs. A) Diagram showing the scheme for the test in (B) and (C). B) Optimization of the concentration and ratio of plasmids in the IUE experiment. Error bars, ± SEM. C) Titration of tamoxifen dosage for an appropriate number of OPCs labeled initially. Error bars: mean ± SEM. Kruskal–Wallis test, *p* = 0.0084. Dunn post‐hoc test, *p < 0.05, **p < 0.01. D) Representative immunofluorescence image showing a single OPC labeled after 48 h of electroporation. CC, corpus callosum. Ctx, cortex. LV, lateral ventricle. Pir, piriform cortex. Str, striatum. Scale bar, 10 µm. E–G) Representative large clone generated by the labeling system. This clone contained 98 cells, and overstraddled both the gray and white matter. G) 3D reconstruction of the clone from (E,F) built by Neurolucida (MBF). The boundary of the anatomical structures was demarcated. P*α*, Pdgfr*α*. Scale bars in (E): 50 µm. H) Scheme for NND model in (I). The box above shows the plasmids electroporated into the brain. Box below shows the genotype of mice. The diagram of the brain displays three possible labeling patterns. I) NND analysis of OPCs labeled by electroporation. The lines represent the cumulative frequency of the NNDs of the labeled OPCs. The green line represents OPCs expressing EGFP only, the carmine line for OPCs expressing EGFP and tdTomato together, and the gray lines show the simulated random data set.

To achieve this goal, we carefully optimized the PB‐reporter system using the *NG2‐Cre^ERT^* transgene to perform clonal analysis with temporal resolution. As described in **Figure** **3**A, we performed IUE at E14.5 in *NG2‐Cre^ERT^* mice, but administrated tamoxifen to newborn pups starting at P3 via subcutaneous injection. The brains were harvested 48 hours after tamoxifen treatment to quantify labeling efficiency. We predicted that both the concentration of PB/PBase vectors and the dose of tamoxifen would affect the labeling efficiency. Therefore, we first optimized the amount and ratio of vectors used for IUE. As shown in Figure 3B, with the same dose of tamoxifen (25 mg kg^−1^), more plasmid DNA resulted in higher labeling efficiency. We eventually chose the DNA concentration that consistently labeled ≈50 cells in the entire brain; that is: 0.5 µg µL^−1^ PBase and 1 µg µL^−1^ PB DNA in combination. Next, we titrated the concentration of tamoxifen, and observed a clear positive correlation between the amount of tamoxifen and the labeling efficiency. We finally decided that 12.5 mg kg^−1^ tamoxifen (one dose) was a suitable dose that consistently labeled 10–20 cells per mouse brain (Figure 3C). As expected, all labeled cells expressed PDGFR*α*  (one representative image is shown in Figure 3D), showing the specificity of labeling OPC lineage cells. Importantly, no EGFP‐labeled OPC was found in the entire brain without tamoxifen treatment (*N* = 5 brains in total), demonstrating the extremely clean background of our clonal analysis system. One representative clone containing 98 cells from dorsal geminal zone‐derived OPCs is shown in Figure 3E–G.

To validate the clonality of sparsely labeled cells, we first performed a time‐course BrdU labeling experiment. We co‐injected BrdU and TAM at P3 and analyzed the brain at 48 and 72 h post TAM injection (Hpi). At 48Hpi, the majority of BrdU‐labeled GFP+ cells were single cells. No BrdU+ GFP+ twin‐spots were detected (Figure S4A, supporting information). We did find some twin spots negative for BrdU, which we believe escaped from BrdU labeling due to the overall low BrdU labeling efficiency. However, at 72Hpi, some GFP+ twin‐spots (which were considered clones based on their distance) could be both labeled by BrdU (Figure S4B, supporting information). Interestingly, the BrdU intensity of the twin spots is often weaker than that of BrdU‐labeled single cells, suggesting a dilution of BrdU during cell division. We did not observe any twin spots containing mixed BrdU‐labeled cells (that is, all were negative or all were positive for BrdU labeling). These data support that the cell clusters close to each other are likely bona fide clones.

To rigorously prove the clonality of our OPC “clones,” we adopted a mathematical simulating modeling termed Nearest Neighboring Distance (NND) by precisely measuring the distance between all labeled cells in a defined brain region (Figure 3H,I). NND has been widely used to validate the true clonality for in vivo clonal analyses of neural stem cells, neural progenitors, excitatory neurons, and inhibitory neurons.^[^ ^38^, ^39^, ^41^, ^42^, ^43^, ^44^ ^]^ In this method, in addition to the PB‐based GFP labeling system, we also introduced an independent clonal labeling system with a different color (tdTomato). The labeling of OPCs by tdTomato was achieved by using a dual recombinase reporter mouse line termed *Rosa26^CAG‐FSF‐LSL‐tdTomao^* (or *Ai65(RCFL‐tdT)‐D* ^[^ ^45^ ^]^), together with a DNA plasmid encoding a codon‐optimized Flippase (FlpO) and the *NG2‐Cre^ERT^* transgene. The specific expression of tdTomato in OPCs depends on the presence of the FlpO plasmid and TAM‐dependent *NG2‐Cre^ERT^* activation (Figure 3H). Therefore, the final dual labeling system included two transgenic alleles (*Ai65* and *NG2‐Cre^ERT^*) and three plasmids (hyperPBase, PB‐GFP vector and CMV‐FlpO). HyperPBase, PB vector, and *NG2‐Cre^ERT^* were used to generate GFP‐labeled OPC clones as described in the previous manuscript, while CMV‐FlpO, Ai65, and *NG2‐Cre^ERT^* were used to generate tdTomato‐labeled OPC clones (Figure 3H and S5, Supporting Information). As multiple plasmids could be transfected into the same cell during electroporation, double‐colored (Y) cells could also be observed (Figure S5C, Supporting Information). Importantly, as the labeling process by distinct colors is independent, if the cell clusters represented true clones, clones containing cells with more than one color are not expected to be seen.

All labeled cells at the time point of 48 h post tamoxifen administration (48Hpi) were carefully recorded, and their coordinates in the brain were precisely calculated after 3D reconstitution of consecutive sections. The NND among all green cells (G‐G), all double‐labeled cells (Y‐Y), or those between any green and double‐labeled cells (G‐Y) were subsequently measured. The NNDs of G‐G cells were significantly shorter than those of simulated random distributions of all labeled cells, indicating that some clones existed among labeled GFP cells (green line in Figure 3I). In contrast, the NNDs of G‐Y cells (magenta line) were comparable to, or even longer than those of simulated random distributions of all labeled cells, suggesting no clonal relationship between any independently labeled green and yellow cells in the same brain region. Notably, as the number of red cells was small, we did not present the NNDs of these cells. Taken together, both the BrdU time course and the NND analysis, together with the independent validation by the MADM model shown below, support the clonality of the OPC clones generated in our models.

Notably, despite the initial labeling occurs in radial glia at E14.5 in our system, generation of OPC clones solely occurs at P3. Therefore, the clonality and sparseness of initially labeled radial glia are unrelated to the clonal analysis at the postnatal stage, as the OPC clones were directly derived from the single OPCs already existing at P3. However, we cannot exclude the possibility that some OPC clones were originally derived from the same radial glia.

### 2.4 MADM‐Based Model Further Validates the OPC Clonality of the IUE Model

**Figure 4:**
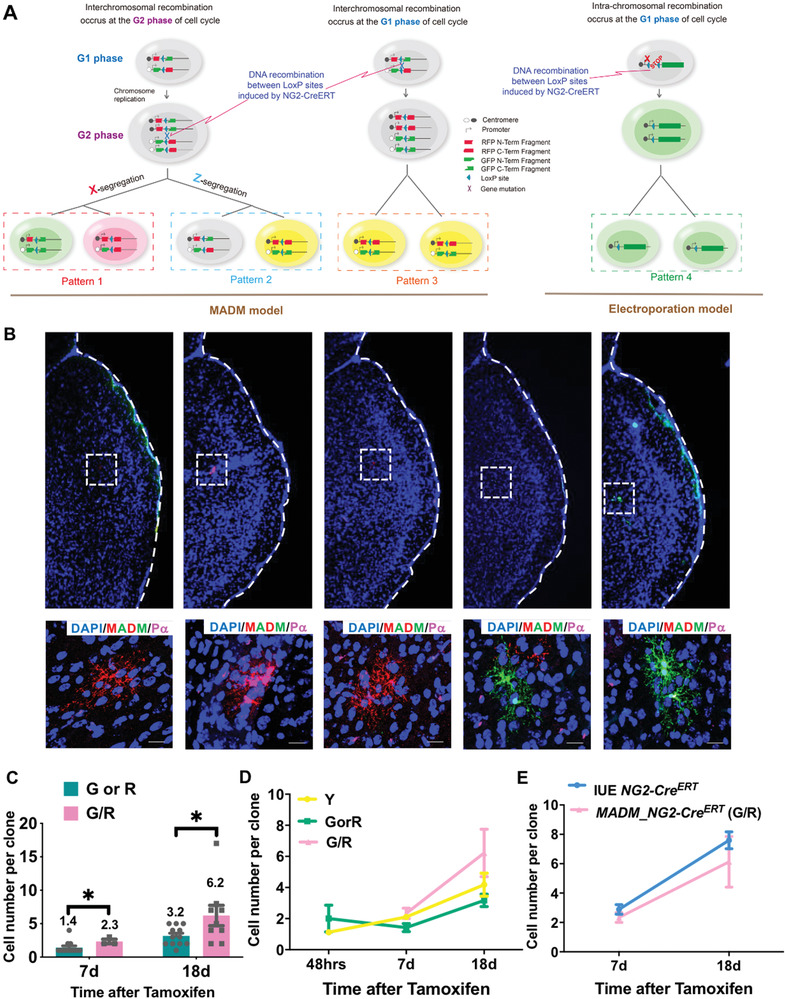
Clonal analysis via the MADM mouse model reproduces the results from the electroporation models. A) Scheme of MADM‐based in vivo clonal analysis model, illustrating how MADM generates cells with distinct colors. The labeling pattern of the electroporation model is also provided. B) Representative images of a MADM OPC clones clone containing both red and green cells. Labeling of cells was achieved by using *NG2‐Cre^ERT^* transgene. The detailed procedure to perform MADM‐based clonal analysis can be found in Section 4. P*α*, Pdgfr*α*. Scale bars, 50 µm. C) The average size of R/G clones was largely 2‐fold of the R or G clones at 7 days and 18 days post injection. R/G clones contained both red and green cells, while R or G clones only contained red or green cells. Error bars: mean ± SEM. Mann‐Whitney test for data of 7dpi. T test for data of 18dpi. One‐tailed. *p < 0.05. D) The average size of clones from the MADM model as indicated. Of note, G or R clones were smaller than others. Error bars: mean ± SEM. One‐tailed Mann–Whitney test for data of 48 hpi. Kruskal–Wallis test for other groups, *p* = 0.0587 for 7 dpi, *p* = 0.1407 for 18dpi. E) The size of R/G clones from the MADM model was comparable to those generated by the electroporation model. Error bars: mean ± SEM. One‐tailed Mann–Whitney test. For the MADM model, there were *N* = 50, 50, and 22 clones for yellow clones at 48 h, 7dpi, and 18dpi, respectively; *N* = 8, 12, and 12 clones for G or R clones; and *N* = 0, 3, and 9 for G/R clones. For the IUE model, there were *N* = 33 and 153 clones at 7 and 18 dpi, respectively.

To further validate the results of the in vivo clonal analysis described above, we utilized a different genetic mouse model, termed Mosaic Analysis with Double Markers (MADM), to perform sparse cell labeling independently. In MADM, two reciprocal chimeric maker cassettes were separately knocked into identical loci on a pair of homologous chromosomes (here Chromosome 11, see also **Figure** **4**A.^[^ ^46^, ^47^ ^]^ Each chimeric marker cassette consisted of a part of the coding sequences of GFP and tdTomato. The N and C terminals of each cassette were separated by an artificial intron in which the LoxP site was positioned. In the G2 phase of the cell cycle, the expression of Cre recombinase (such as *NG2‐Cre^ERT^* used here) triggers inter‐chromosomal recombination between the LoxP sites and restores the expression of functional GFP and tdTomato. If the sister chromatids are assigned to the two daughter cells following the pattern of so‐called X‐segregation, a pair of GFP‐ and tdTomato‐labeled cells will be generated (Figure 4A, Pattern 1). However, if the sister chromatids are sorted following the pattern of so‐called Z‐segregation, a pair of unlabeled and double color (yellow) labeled daughter cells will be produced (Figure 4A, Pattern 2). Of note, recombination can also directly occur at the G1 phase of the cell cycle or in postmitotic cells, where the yellow cells are generated (Figure 4A, Pattern 3). Importantly, this last pattern of labeling is phenotypically equal to the labeling pattern of the IUE‐based model described above (compare Patterns 3 and 4 in Figure 4A), where the mother OPCs were directly labeled.

As the efficiency of inter‐chromosomal recombination between loxP sites is extremely low, MADM is suitable for generating sparsely labeled cells. Because of this feature, MADM has been successfully used to perform clonal analysis of multiple cell types including embryonic neural progenitors and adult hippocampal stem cells.^[^ ^38^, ^39^, ^44^, ^46^, ^48^, ^49^, ^50^ ^]^ In addition, the unique feature of simultaneously producing paired green and red cells offers MADM the power to dissect the division pattern of progenitor cells in vivo. We have previously used this system to examine the division pattern of OPCs after acquiring glioma‐relevant mutations.^[^ ^15^, ^51^ ^]^ Therefore, we used tamoxifen‐inducible *NG2‐Cre^ERT^* to induce mitotic recombination directly in OPCs at P3 and examine the clonality of labeled cells. We confirmed that only OPC‐lineage cells could be labeled in the system and that the labeling was fully dependent on the presence of tamoxifen (data not shown). As predicted, red, green, and yellow cells could be all detected in the *MADM_NG2‐Cre^ERT^* brain (Figure 4B and not shown). However, the labeling was extremely sparse, with only ≈50 cells detected in each brain after 48 hours of tamoxifen administration.

Next, we studied the cellular composition of each clone in the MADM model. Most clones contained only yellow cells, suggesting that they derived either from one double‐labeled daughter cell after G2‐Z segregation (Pattern 2), or directly from G1 mother OPCs (Pattern 3). We also found that “twin‐spot” clones contained both red and green cells (G/R clones, see also Figure 4B as an example). These clones were most likely derived from a single unlabeled mother OPC after G2‐X segregation (Pattern 1). Interestingly, we also found clones containing only green or red cells (G or R clones). We reasoned that these single‐color G or R clones were derived from one daughter cell immediately after the G2‐X segregation, and that the other daughter cell died immediately after initial cell division (one cell died after division of pattern 1). If this speculation is correct, we should expect the size of R/G clones to be 2‐fold of the R or G clones, which was exactly what we saw (Figure 4C,D). At both timepoints analyzed (7 dpi and 18 dpi), the sizes of R/G clones were 2.3 and 6.2, while the sizes of R or G clones were 1.4 and 3.2 respectively. The average size of double‐colored (yellow) clones was between that of the R/G clones and single‐color clones (Figure 4D). This was expected, as double‐colored clones contained both G0‐derived (the size should be comparable to R/G clones, pattern 3) and G2‐Z segregation clones (the size should be comparable to R or G clones, pattern 2). Importantly, the size of R/G clones was comparable to that generated by the IUE model (Figure 4E) described above (pattern 4 in Figure 4A). Therefore, these data further validate the bona fide clonality of the cell clusters labeled in our IUE model described above.

### 2.5 OPC Clones Derived from the Dorsal Germinal Zone Expand Continuously, but those from the Ventral Zone Vanish Eventually

**Figure 5:**
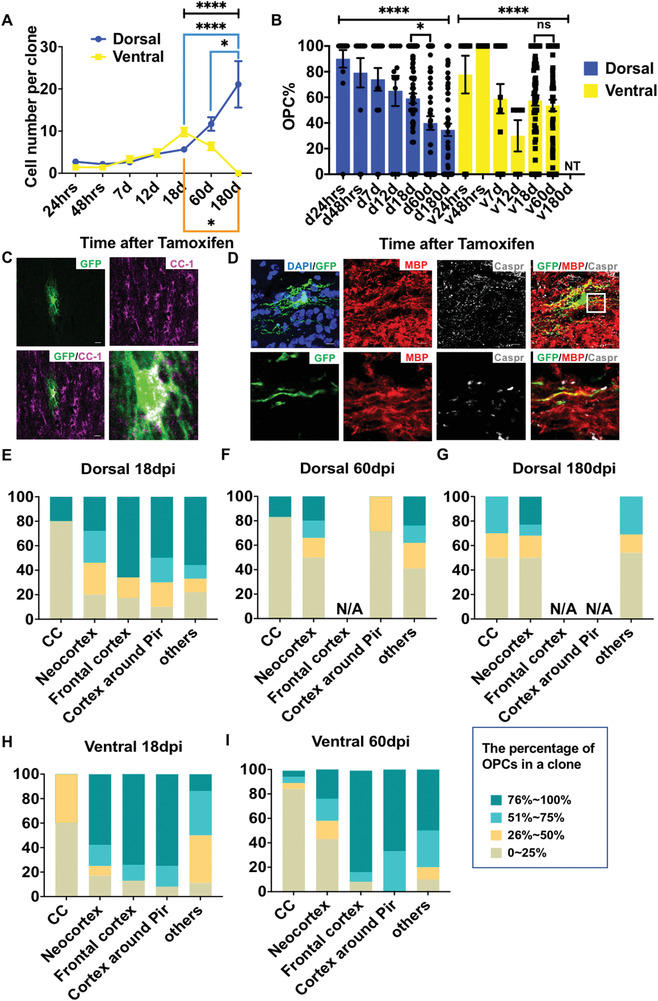
The proliferation and differentiation potentials of OPC clones derived from dorsal and ventral germinal zones. A) The average size of clones as indicated. Of note, OPC clones from the ventral origin eventually vanished from the brain. Error bars: mean ± SEM. Two‐way ANOVA followed by Tukey post‐hoc test, *p < 0.05, ****p < 0.0001. B) The proportion of OPC in each clone. Error bars: mean ± SEM. Kruskal–Wallis test followed by Dunn post‐hoc test. *p < 0.05, ****p < 0.0001. ns, not significant. C) Representative images of an oligodendrocyte derived from ventral OPC expressing CC1, the differentiated oligodendrocyte marker. Scale bars, 10 µm. D) Representative images of an oligodendrocyte derived from ventral OPC expressing MBP, the myelinating oligodendrocyte marker and the staining of Caspr, which is the marker protein of paranodes. Scale bars, 10 µm. E–I) The OPC percentage in each clone from different brain structures. N/A, not applicable, which means no clone was found in these areas of those groups. For dorsal groups, there were *N* = 15 clones for 24 h, *N* = 12 for 48 h, *N* = 16 for 7dpi, *N* = 12 for 12 dpi, *N* = 81 for 18 dpi, *N* = 60 for 60 dpi, and *N* = 61 for 180 dpi. For ventral groups, there were *N* = 9 clones for 24 h, *N* = 27 for 48 h, *N* = 17 for 7 dpi, *N* = 5 for 12 dpi, *N* = 72 for 18 dpi, and *N* = 89 for 60 dpi.

After establishing the clonal method, we next examined the developmental trajectory of individual OPCs by quantifying the clones at distinct time points after tamoxifen administration. The clones were obtained following the procedure described in the Experimental Section. OPC clones derived from both the dorsal and ventral germinal zones continuously expanded in the first two weeks after initial labeling (**Figure** **5**A). Surprisingly, despite the clones from the dorsal origin continuing to expand beyond this timepoint, the average size of the ventrally derived clones started to drop off and completely disappeared at 180 days post tamoxifen injection (dpi). These results at the clonal level are consistent with the population level study (Figure 2G), and strongly suggest that OPCs derived from distinct germinal zones are heterogeneous in their capacity for self‐renewal. Intriguingly, the impaired self‐renewing competence of ventrally derived OPCs only became prominent after the mice reached maturation. At an earlier stage (such as before 18dpi), however, the average rate of clonal expansion for ventrally derived ones was comparable to or even slightly higher than that of dorsally derived ones (Figure 5A), which is consistent with the results based on Ki67 staining at the population level (Figure 2F).

Next, we quantified the composition of the cell types in each clone. We confirmed that all clones contained only the OPC lineage cells. We did not find astrocytes or neurons in any of the clones examined (data not shown), confirming the previous opinion at the clonal level that OPCs are largely unipotent in the postnatal brain under normal physiological conditions.^[^ ^52^ ^]^ We then calculated the percentage of OPCs and oligodendrocytes in each clone to examine the differentiation potential of individual OPCs at the clonal level. Interestingly, although dorsally derived clones expanded continuously during development, the percentage of OPCs in each clone conversely decreased. In contrast, the decrease in OPC composition in ventrally derived clones was not as striking as that in their dorsal counterparts (Figure 5B). This observation suggests that dorsally derived OPCs exhibited stronger potential to self‐renew, but paradoxically had a higher tendency to differentiate when compared to the ventral clones, which is highly consistent with the results seen at the population level (Figure 2E). Immunostaining by mature oligodendrocyte markers such as CC1 and MBP, in addition to Caspr, the marker protein of paranodes, confirmed that ventrally derived OPCs can undergo terminal differentiation into myelinating oligodendrocytes (Figure 5C,D). Given that the size of clones derived from the ventral germinal zone decreased and eventually disappeared, these data also raise the possibility that mature oligodendrocytes derived from the ventral germinal zone are more prone to death than their dorsal counterparts.

### 2.6 Distinct Brain Anatomical Structures May Play Different Roles in OPC Self‐Renewal

To examine whether the distinct anatomical structures of the brain affected OPC differentiation at the clonal level, we quantified the OPC percentage in each clone according to its location in the brain (Figure 5E‐‐I). In general, regardless of developmental origin, clones in the white matter contained more differentiated oligodendrocytes than those in the gray matter. For instance, at 18dpi, the majority of clones in the CC were highly differentiated (containing less than 25% OPCs). In stark contrast, most clones in cortical regions remained undifferentiated at this stage (compare Figure 5E,H). These results are in good agreement with previous lineage‐tracing studies that OPCs are prone to differentiation in the white matter,^[^ ^2^, ^23^, ^42^ ^]^ therefore supporting the idea that local brain environments may play a role in OPC homeostasis. However, we also found that in certain brain structures such as the piriform cortex, dorsal and ventral clones exhibited very different differentiation potentials, particularly at the adult stage. Although most dorsal clones were highly differentiated in the piriform cortex, ventral clones in the same regions were largely undifferentiated (compare the fourth columns between Figure 5E,H, and those between Figure 5F,I). Therefore, our data strongly support the notion that developmental origins play a role in regulating the homeostasis of OPCs at later developmental stages. Of note, because of the relatively low percentage of actively proliferating cells within individual brain regions, we could not compare the proliferation rate of different origin‐derived OPCs in the same region.

Intriguingly, we also observed some OPC clones in the olfactory bulb (Figure S6A, Supporting Information), although quite rarely and only in the ventral IUE model. This unique population of OPCs has been described previously.^[^ ^40^ ^]^ Our data further suggest that at least some OPC clones in the OB are derived from the ventral germinal zone during the early developmental stage. We also frequently found clones that straddled multiple brain structures, such as distinct cortical layers within gray matter, or both the gray and white matter. For instance, some clones occupied both the CC and deep cortical layers (layer VI) (Figure S6B, Supporting Information). Therefore, our in vivo clonal analysis confirmed that the brain's structure did not restrain the expansion of OPC‐lineage cells derived from single progenitors.

### 2.7 Clonal Analysis Unravels the Unique Unitary Production of OPC Clones in the Early Stage

**Figure 6:**
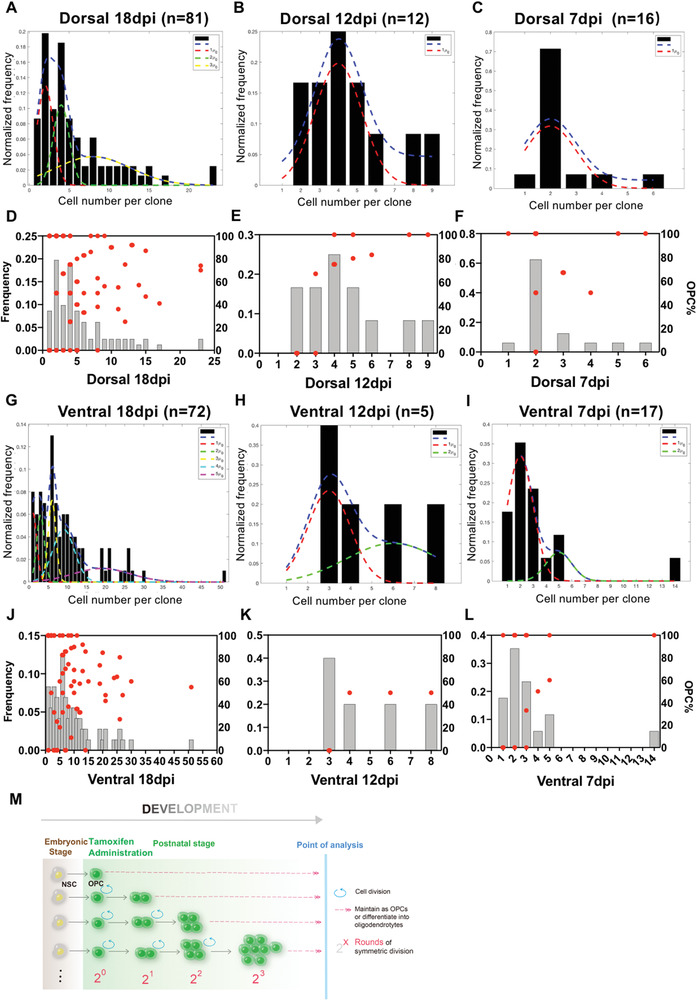
OPC clones exhibited an integer multiple of the Gaussian unit in the early stage. A–C) Gaussian Fitting of the distribution of the sizes of dorsally derived clones at A) 18 dpi, B) 12 dpi, and C) 7 dpi. The black columns represent the frequency of clone sizes. The dotted lines show the simulated distribution with the integer number as indicated. D–F) The percentage of OPCs in each clone was aligned with the clone size. The gray columns represent the frequency of clone sizes same as that in (A–C). The red dots represent the OPC percentage in each clone. G–I) Gaussian Fitting of frequency distribution of the sizes of ventrally derived clones at G) 18 dpi, H) 12 dpi, and I) 7 dpi. J–L) The percentage of OPCs in each clone was quantified according to the clone size. *N* number was indicated above the graphs. M) The deduced dividing pattern of dorsally derived OPCs at early stage based on the data from (A–C).

The histogram of clone sizes at P21 (18dpi) revealed that the overall distribution of clonal size appeared nonrandom. Dorsally derived clones exhibited an integer multiple of the Gaussian unit of 2*^n^* (i.e., 1, 2, 4, 8) (**Figure** **6**A). Therefore, we reasoned that the dorsal OPC clones mainly underwent symmetric divisions before weaning. Should this be the case, we should expect to see that at an earlier stage before P21, integer multiples of the Gaussian distribution are still maintained, but only smaller size of clones should be observed. Indeed, when we examined the histogram of clone sizes at P15 (12dpi), the clonal size centered on the mean value of 4 (2^2^). At P10 (7dpi), the clonal size centered around 2 (2^1^) (Figures 6B and 6C respectively). These observations strongly suggest that OPCs originating from the dorsal germinal zones mainly underwent symmetric divisions before the weaning age. However, after this time point, the unitary distribution of clones became blurred, likely due to the non‐synchronized cycling of individual clones or random cell death at a later stage (Figure S7A,B, Supporting Information).

Next, we asked whether the dorsal clones are destined to grow exponentially, or whether they intrinsically possess the capacity to divide only certain times. We reasoned that if the distribution of integer multiples of the Gaussian unit of 2*^n^* resulted from the snapshots of clones underdoing exponential growth, the clones of the Gaussian unit of 2^2^ should be the precursor of those of 2^3^. If this were the case, all cells in clones of 2^2^ must be OPCs to be able to further divide at least once. However, when we analyzed the OPC percentage along the clone size, we found that regardless of clonal size, both OPCs and oligodendrocytes could be found in a large proportion of clones of 2^2^ and 2^3^ (Figure 6D–F). These results suggest that some OPCs were destined to divide twice, but others divided three times in a symmetric way before differentiation (as shown in Figure 6M). Therefore, we conclude that dorsally derived OPCs undergo symmetric division in a “quantum” fashion, at least in the early postnatal stage.

It should be mentioned that, in our mathematical models, we only considered the simplest scenario, presuming that no cell death occurred, since the pattern of exponential expansion most likely supports all cells remaining alive in the clones during the early stage of development (<P21, 18dpi). Indeed, when compared with the MADM data, the clonal size was comparable to those containing both green and red cells (R/G) rather than those containing single red or green cells (R or G), with the latter indicating that one of the twin spots died at earlier time (see Figure 4C‐E). These results further supported the lack of cell death in the majority of clones analyzed before P21. However, we could not exclude the possibility that cell death occurred in some clones, since the size of the clones became more random as they developed (Figure S7), suggesting that the cells randomly died as the clones grew.

Surprisingly, when we analyzed the histogram of ventrally derived clones in a similar way, we found a quite distinct distribution pattern of the clonal size. Although they also exhibit an integer multiple of the Gaussian unit, at P21, ventrally derived clones exhibited an integer multiple of the Gaussian unit of 3n (namely 1, 3, 6, 9) (Figure 6G). Because of the relatively small sample size of early‐stage clones (Figure 6H,I), we are unable to convincingly deduce how such clonal size of linear Gaussian units formed, although mathematical deductions suggest a different cycling time between the twin cells after each division might produce such a pattern. Regardless of this possibility, we could conclude that the patterns of clonal expansion and cell division from dorsal and ventral germinal zone‐derived OPCs are different.

### 2.8 OPC Clones Can be Classified into Five Subtypes Based on their Spatial Organizations

**Figure 7:**
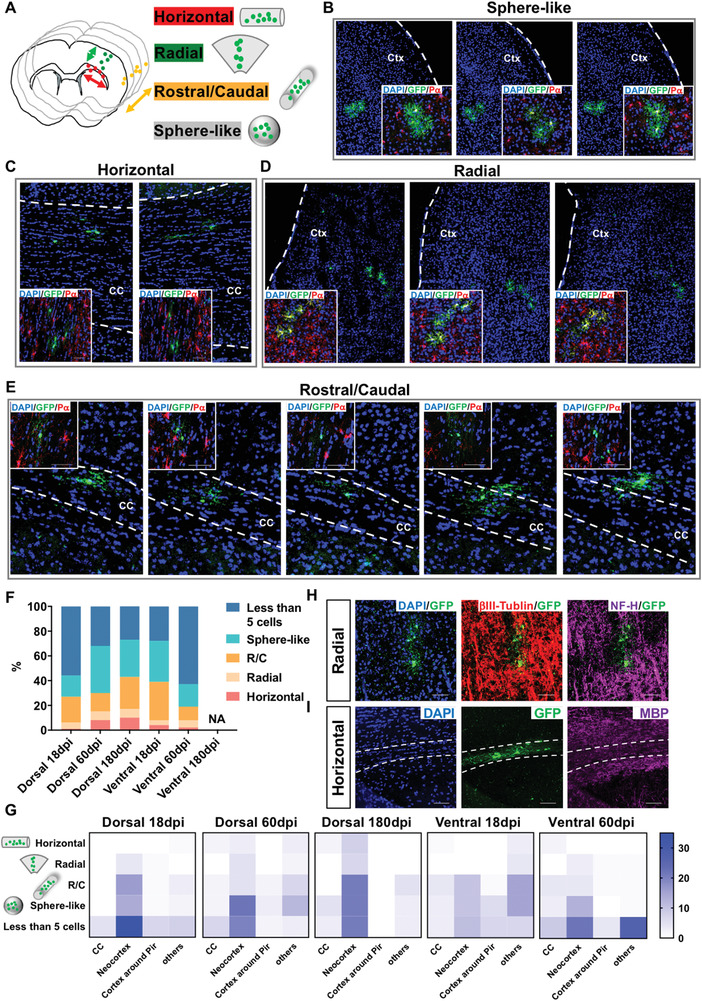
OPC clones can be classified as five subtypes based on their spatial orientations. A) Models of four subtypes of OPC clones based on their shapes. B–E) Representative immunofluorescent images of the four subtypes of clones as indicated. The insets show the zoom‐in image of the clone. Ctx, cortex. CC, corpus callosum. Scale bars, 50 µm. F) The abundance of 5 subtypes of clones along their development. NA, not applicable as no clones were found. G) The abundance of 5 subtypes of clones within different brain structures. CC, corpus callosum. Pir, piriform cortex. See also Videos S1–S4, Supporting Information for the 3D reconstruction of each representative subtype of clones. H) Representative images of a Radial‐subtype clone in the cortex. The staining of neuron‐specific *β*III‐Tubulin and Neurofilament‐H delineates the trend of the axons. I) Representative images of a Horizontal‐subtype clone located in the CC. The staining of MBP delineates the distribution of the myelin.

To help understand the pattern of clonal expansion of individual OPCs, we next analyzed the shape of clones based on their developmental origins, location within brain structures, and the developmental time points. We performed consecutive brain sections and reconstructed the position of each cell within the clone, with the boundary of major brain structures as a reference. We found that all clones could be generally classified into four types based on their shapes and orientations: horizontal, radial, rostral/caudal, and sphere‐like. However, the shape of clones containing less than five cells could not be defined (**Figure** **7**A–E, and Video S1–S4, Supporting Information).

When analyzing the subtypes of clones throughout development, we found that sphere‐like clones made up majority of the clones regardless of the developmental timepoint or origin. In contrast, radial clones were rare. Interestingly, we found that in the dorsal IUE model, the abundance of clones with less than five cells continuously decreased with age. Instead, the abundance of all four clones increased accordingly (Figure 7F). These observations suggest that clones with less than five cells grew over time and contributed to the pool of clones with defined shapes. Interestingly, in the ventral model, the percentage of clones with less than five cells increased during development, and clones with defined shapes, such as R/C and radial ones, became less abundant in the mature brains (Figure 7F). These data are consistent with the analysis of clonal size (Figure 5A), and suggest that the size of ventral zone‐derived OPC clones reduced after the mice matured.

Finally, we examined whether the shape of the clones was associated with certain brain structures. We focused on three representative structures, namely: the CC, neocortex, and the posterior portion of the cortical region. We found that horizontal clones were most frequent in the CC, although in the dorsal model, they could also be found in some posterior cortical regions. In contrast, radial clones could only be detected in cortical regions (Figure 7G). These data suggest that local brain structures may affect the orientation of OPC clones, which influences their ability to expand. To further validate this hypothesis, we co‐stained radial‐shaped clones in the cortical region with neurofilament markers NF‐H and *β*III‐tubulin, which marked the orientation of neuronal processes in the same brain region. Indeed, we found that the orientation of the radial clones was well aligned with that of the axons (Figure 7H). Similarly, horizontal clones were distributed along the corpus callosum (CC), as visualized by MBP and DAPI staining (Figure 7I). Therefore, it appears that the shape of an OPC clone is determined, at least partially, by the orientation of the axons and the amount of space available between axons within the regions where the clone resides, which is consistent with a previous study explaining the arrangement of oligodendrocytes relative to axons and myelination.^[^ ^53^ ^]^ Such an arrangement might be critical for clone‐derived oligodendrocytes to readily myelinate local axons.

### 2.9 *NF1* is a Critical Factor to Reverse the Cell Fate of Ventrally Derived OPCs

What controls the fate of the ventrally derived OPCs? We and others have previously shown that Neurofibromatosis 1(*NF1*, a member of the GAP family that negatively regulates the activity of Ras) plays an important role in OPC homeostasis. Knockout of *NF1* in OPCs or neural progenitors resulted in dramatic overexpansion of OPCs at the population level.^[^ ^54^, ^55^, ^56^, ^57^ ^]^ Therefore, we examined whether *NF1* deficiency could prevent the loss of ventrally derived OPCs during development.

**Figure 8:**
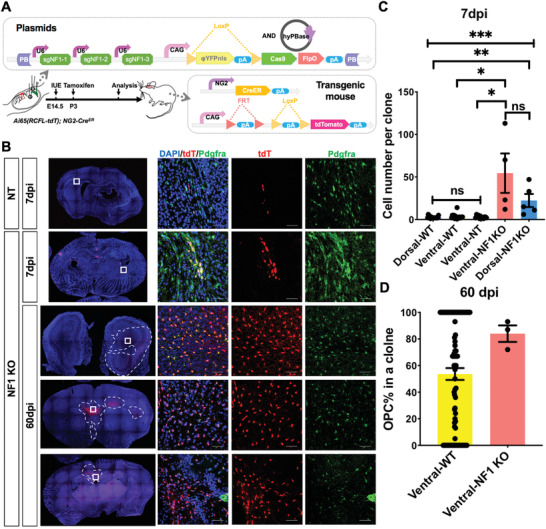
*NF1* KO can convert the death of ventrally derived OPCs. A) Configuration of the gene knocking‐out approach mediated by the PiggyBac transposon reporter system and CRISPR‐Cas9 in this study. The expression of cell‐type specific recombinase will remove the ΦYFP coding sequence along with the stop signals (LSL) and result the expression of Cas9 and FlpO on the PB reporter plasmid. The Cre and FlpO recombinases will remove the double stop signals (FSF‐LSL) to label the cells through expression of tdTomato. At the same time, Cas9 and sgRNA controlled by U6 promoters will edit the gene of interest. B) Representative immunofluorescent images of cells electroporated with plasmids carrying tandem sgNT (Non‐targeting sgRNA) or tandem sg*NF1* at the indicated age. The white squares in the brain slice map indicate the location of zoom‐in images on the right. Scale bars, 50 µm. C) The average size of clones as indicated. WT means the cells labeled by the previous system. Error bars: mean ± SEM. Kruskal‐Wallis test followed by Dunn post‐hoc test. *p < 0.05, **p < 0.01, ***p < 0.001. ns, not significant. D) The proportion of OPC in each clone as indicated. Error bars: mean ± SEM. One‐tailed Mann–Whitney test. For the *NF1* KO model, there were *N* = 4, 3 clones for ventral *NF1* KO clones at 7 and 60 dpi, respectively; *N* = 5 clones for dorsal *NF1* KO clones at 7 dpi; and *N* = 12 clones for ventral non‐targeting clones. For the WT model, there were *N* = 16, 89 clones at 7 dpi (both dorsally and ventrally derived) and 60 dpi, respectively.

For this purpose, we developed a novel system by combining the electroporation‐based clonal analysis technique with a CRISPR‐Cas9‐based gene knockout system to generate wildtype or *NF1*‐/‐ OPC clones (**Figure** **8**A). In the system, a cassette encoding a LoxP‐Stop‐LoxP‐Cas9‐2A‐FlpO, along with an array of sgRNAs specific to the gene of interest, was positioned in the PB vector. In combination with the Cre‐FlpO dual recombinase reporter *Ai65* and *NG2‐Cre^ERT^* (as shown in Figure 3H for the NND analysis), this system can specifically generate genetically manipulated OPC clones and trace these with high fidelity. To increase the efficiency of gene editing, three sgRNAs targeting distinct sites of *NF1* were introduced into the targeting vector. As a non‐specific control, an array of sgRNAs targeting the sequence not existing in mice was used to generate WT OPC clones. The specificity of these sgRNAs to knock out *NF1* was confirmed by RNA sequencing (Figure S8, Supporting Information).

As shown in Figure 8B,C, in the absence of *NF1*, both ventrally and dorsally derived OPCs expanded as early as 7dpi. Quantitative analysis confirmed a significant increase in clone size compared to the nonspecific control (NT) or WT clones derived from both ventral and dorsal origins, indicating that *NF1* knockout promotes OPC proliferation (Figure 8C). It should be noted that as the average size of NT clones is comparable to that of WT OPCs produced by the method described in Figure 3 (compared to the first three columns in Figure 8C), we concluded that the clones generated by this system did not affect the development of normal OPCs in vivo. In stark contrast to that shown in Figure 5A, wherein the size of ventrally derived WT OPCs decreased, the size of their *NF1* ^−/−^ counterparts continued to expand (Figure 8B,C). Interestingly, not only did the size of the ventral *NF1* ^−/−^ OPC clones drastically increase, but also the differentiation potential into oligodendrocytes was found to be inhibited at the later developmental stage (Figure 8D). In summary, we conclude that *NF1* deficiency can not only promote the proliferation of OPCs, but also reprogram the fate of ventrally derived OPCs and prevent their distinction at later developmental stages.

## 3 Discussion

In this study, we aimed to address the question of whether developmental origins contribute to the phenotypic heterogeneity of OPCs in the adult brain. Taking advantage of the single‐cell resolution fate‐mapping system developed in this study, we traced the fate of early OPCs derived from either the dorsal or ventral germinal zone at both the population and clonal levels. Not only did we confirm that OPCs were largely unipotent regardless of their origins, but also revealed that the developmental trajectory of OPCs in mature brains was affected by their developmental origins, wherein OPCs originating from the dorsal germinal zone possessed the ability to expand throughout development, while those from the ventral side eventually vanished. Furthermore, in vivo clonal analysis revealed that the early division patterns of OPCs could also be affected by their developmental origins. We also provide data showing that NF1 can reprogram the fate of ventral germinal zone‐derived OPCs.

### 3.1 In Vivo Clonal Analysis and Its Application in OPC Biology

It has been increasingly appreciated that cells, which have been previously viewed as a homogeneous population, can exhibit very distinct behavior in vivo. Deciphering the developmental origin of such heterogeneity of a defined cell type, and the association of such heterogeneity with the potential diversity of cellular functions, are important questions that still need to be answered in the field of developmental biology.

While the availability of new techniques such as single‐cell transcriptome sequencing and in situ sequencing, have greatly advanced our understanding of cellular heterogeneity in vivo, we are still far away from fully understanding the developmental basis of cellular heterogeneity in most cell types. In vivo clonal analysis is an important approach, which not only helps depict the lineage map of certain progenitors, but also facilitates the understanding of the cellular heterogeneity of the desired cell type at the single cell level, as the developmental trajectory of each progenitor can be precisely defined temporospatially. Taking advantage of its unprecedented cellular resolution and noninvasive nature, in vivo clonal analysis has been widely used to study the development and heterogeneity of a variety of stem cells/progenitors in the brain, especially those that cannot be successfully recovered by single‐cell transcriptome approaches.^[^ ^23^, ^38^, ^39^, ^40^ ^]^

OPCs are one of the earliest cell types in the CNS that were studied using clonal analysis. Before the availability of genetic labeling approaches, Raff and his colleagues established a pioneering technique by performing ex vivo clonal analysis of purified OPCs from optical nerves, which led to important findings in the field of OPC biology.^[^ ^58^, ^59^, ^60^, ^61^ ^]^ Although some of these findings may only be applied to cultured systems, many have been validated by later in vivo studies using genetic approaches. For instance, by using the *NG2‐Cre^ERT^* transgene, Zhu et al. sparsely labeled OPCs and observed the presence of development‐dependent OPC‐to‐astrocyte trans‐differentiation.^[^ ^62^ ^]^ They also revealed that OPCs from different brain regions and/or developmental time points possessed distinct patterns of homeostasis to balance their proliferation and differentiation. Using a novel multicolor labeling system termed Startrack, Garcia‐Marques et al. confirmed that some OPCs can expand throughout the life and generate huge clones containing >300 cells.^[^ ^40^ ^]^ However, as the expression of marker genes in this study was controlled by the hGFAP promoter, which was downregulated after OPC differentiation, the composition of mature oligodendrocytes in these clones was undetermined. In general, these previous studies highlighted the heterogeneity of OPCs in vivo.

### 3.2 Developmental Origins and OPC Heterogeneity

By further dissecting the developmental origins of OPCs, we performed clonal analysis of dorsal and ventral germinal zone‐derived OPCs. This allowed us to directly examine the relationship between the developmental origin and heterogeneity of OPCs. Surprisingly, we found that dorsally derived OPCs possessed the capacity to continuously expand, while those from the ventral side eventually disappeared. This finding directly proved that the developmental origin indeed plays a role in the phenotypic heterogeneity of OPCs at later stages.

Using a set of elegant genetic tools, Richardson and his colleagues defined three waves of OPC development, and found that OPCs originating from MGE‐APE died out in the neonatal brain. However, those from the LGE and cortical regions persisted in the adult brain.^[^ ^31^ ^]^ While our studies at the clonal levels partially support the conclusions from this previous work, there is a key difference in terms of the fate of ventrally derived OPCs. Our results support that ventrally derived OPCs, which likely included those from LGE/CGE, also died out eventually in the adult brain. In contrast, this subpopulation was reported to persist in the adult brain in the study by Kessaris et al., wherein they mapped the fate of the progenitors in LGE/CGE by using *Gsh2 (or Gsx2)‐Cre* transgenes.

Several possibilities may explain this discrepancy. First, the LGE/CGE precursors that could produce OPCs persistent to the adult stage might not have been targeted by our IUE methods. While we could not fully exclude this possibility, we believe this may not be the main reason. We performed a significant number of IUEs, and in each embryo, the electroporation orientation could have been slightly different. Therefore, it is highly unlikely that such a precursor would have escaped our tracing system if it made up a significant portion of the cells analyzed. Second, in the current study we only traced the fate of OPC clones generated at or earlier than P3. Thus, it is possible that some OPCs might be continuously produced by LGE/CGE precursors later than this stage, and those OPCs possess the capacity to persist into the adult stage. However, the results from our *NG2‐Cre* IUE model strongly argue against this possibility, since all OPCs from the initially targeted LGE/CGE precursors were labeled upon analysis. In the *NG2‐Cre* ventral IUE model at the population level, we still could hardly detect labeled OPC‐lineage cells in adult brains. The last explanation is that the *Gsh2‐Cre* transgene used by Kessaris et al. may directly label OPCs regardless of their origin. Indeed, the transcriptome data of Barres group^[^ ^63^ ^]^ and those from our own laboratory (Figure S9, Supporting Information) clearly showed that OPCs by themselves express Gsx2. Further studies using more specific (or temporally controllable) genetic tools are warranted to fully address the question of whether OPCs in adult brains are solely contributed by dorsally derived precursors. Nevertheless, our study emphasizes the importance of taking into considerations of developmental origins for OPC‐based cell therapies with maximal efficiency.

Our data based on clonal analysis are generally consistent with previous studies, and also reconcile some previous conflicts. Tripathi et al. observed that there were similar numbers of both dorsally and ventrally derived OPC lineage cells at postnatal in the dorsal and dorsolateral funiculi at cervical levels, but dorsally cells outcompete their ventral counterparts and dominate the dorsal tracts at the young adult stage. Despite a different angle of study of the remyelination process after acute demyelination, Crawford et al. revealed that dorsally derived OPCs have a more positive response to demyelination, outperform their ventral counterparts in recruitment and differentiation, and dominate remyelination even in the ventral zones of the CNS. In particular, although both dorsal and ventral OPCs contributed to myelination in the corpus callosum, only those originating from the dorsal origin efficiently took part in the remyelination process. In addition, when dorsally derived OPCs were genetically depleted artificially, OPCs from the ventral germinal zone expanded and dominated the neocortex at the beginning, while the dorsally derived OPCs recovered and outcompete them soon after. Therefore, these previous findings not only parallel our clonal analysis data and support the notion that the cellular behavior of adult OPCs is affected by developmental origins, but also suggest that the ability of OPCs of dorsal origin to expand consistently is important for the CNS to maintain homeostasis and normal functions, and likely also plays important roles during the remyelination process under certain pathological conditions.

Our study also reconciles previous findings. Although the above‐mentioned studies strongly suggest that distinct origin‐derived OPCs are heterogenous, one recent study based on the single‐cell sequencing technique^[^ ^25^ ^]^ argues that OPCs are homogenous at the early developmental stage regardless of their origin. Our clonal analysis results revealed that at an earlier stage, the difference between dorsal and ventral OPCs was minimal in terms of clonal size. However, this difference became prominent after the wean age. Therefore, despite the developmental origin profoundly affecting the eventual fate of OPCs, the effect only starts to appear at a later stage of development, which likely occurs through an epigenetic mechanism that is yet to be determined.

### 3.3 Cellular Clocks in OPCs Control Their Proliferation and Death

It has been previously proposed that there exists a precise intracellular molecular “clock,” which intrinsically determines how many times an OPC can divide before differentiation.^[^ ^64^, ^65^ ^]^ The evidence to support this hypothesis was taken mainly from the in vitro clonal analysis of cultured OPCs, where the researchers found that dividing OPCs were only capable of responding to environmental cues to exit the cell cycle and differentiate into mature oligodendrocytes only after sufficient rounds of division.^[^ ^65^, ^66^ ^]^ However, the molecular basis of such a molecular “clock” is poorly characterized. Furthermore, it is debatable whether such intracellular clocks exist in vivo.

Our in vivo clonal studies, the analysis of a large number of clones at distinct time points support the existence of such cellular “clock” in vivo, at least during the early developmental stage. The histogram of clone sizes before the wean age revealed that dorsally derived clones exhibited an integer multiple of the Gaussian unit of 2*^n^* (Figure 6A), supporting that dorsally derived juvenile OPCs mainly undergo symmetric division. Interestingly, regardless of the clonal size, both OPCs and oligodendrocytes could be found in a large portion of clones of 4 and 8 cells (Figure 6D). These results strongly suggest that some OPCs are destined to divide twice, but others divide three times in a symmetric way before differentiation. These results are nicely reminiscent of previous in vitro studies, thereby supporting the existence of an intrinsic “clock” to control the rounds of division at least for some OPCs.

One puzzling observation is that ventrally derived OPCs exhibit an integer multiple of the Gaussian unit of 3n. However, we still do not understand how such clonal distribution is formed. Further studies are warranted to fully decipher this interesting phenomenon. However, the current study supports the idea that the intrinsic cellular “clock” exists in both dorsally and ventrally derived OPCs but with distinct “clicking speeds.”

Another intriguing observation is that, while OPCs from dorsal and ventral origins exhibited distinct fates in the mature brain, their behaviors were indistinguishable at younger ages in the studies of both population and clonal levels. This observation is consistent with a recent single‐cell study, wherein the authors reported that dorsally and ventrally derived OPCs isolated from the neonatal mouse brain exhibited no difference at the transcriptome level.^[^ ^8^ ^]^ Therefore, OPCs seemingly remember their origins and exhibit differences only when timing comes. We believe that the intrinsic mechanism must, at least partially, regulate this process, because OPCs in the same locations but from distinct origins exhibited distinct fates (Figure 5). It was previously reported that p57Kip2 might be a key player in controlling the “clock” of OPCs in vitro.^[^ ^67^ ^]^ It would be highly interesting to determine whether the expression of p57Kip2 is different in OPCs from distinct origins. Notably, environmental cues could also play roles in determining the fate of OPCs in the adult brain. Indeed, clonal analysis revealed a clear relationship between the differentiation potential and expansion pattern of OPCs in certain brain structures. Therefore, it is very likely that both intrinsic and exterior cues control the fate of OPCs during development in a combinatorial manner.

### 3.4 Advantages and Limitations of the In Vivo Clonal Analysis

In our IUE system, the PB vectors used to label OPCs were designed to execute distinct functions as modules. In the current system, we mainly used the system to introduce lineage markers and perform cell fate studies. It is easy to modify the current system to perform gene knockout (by using the CRISPR/Cas9 system) in any cell type beyond OPCs by using the appropriate Cre drivers. Furthermore, it is possible to perform lineage tracing at the clonal level, as well as genetic perturbations simultaneously in the same cell, when combining all of these modules in the same vector, as shown in Figure 8. In combination with the ability to quickly generate a large number of mouse models within a short period of time, we expect the system to be a powerful tool to study pertinent questions in the field.

As a tool, the IUE system developed in this study also has some limitations. First, owing to the constraints of the system, IUE can only be performed on mouse embryos within certain development time windows (≈E14–E15). Therefore, it may be unable to target cells earlier than this time window. However, when combined with a high‐resolution noninvasive imaging system, such as an ultrasound‐guided technique,^[^ ^68^ ^]^ an earlier timing of IUE could be possible. Second, because of the limited resolution of targeting orientation, the current methods may not cover all the cells of targeted regions, nor precisely distinguish smaller brain regions adjacent to each other, such as the LGE and MGE. To overcome these constraints, more specific Cre drivers could be involved in further enhancing the specificity of the targeted cells.

### 3.5 Concluding Remarks

In summary, by developing a flexible in vivo clonal analysis tool, we have showcased the importance of in vivo clonal analysis to study the heterogeneous features of OPCs and revealed that developmental origins play a role in determining the fate of OPCs in the adult brain. We also provided evidence that intrinsic “clocks” likely exist in OPCs to control their expansion and death throughout life. Further studies are underway to decipher the molecular mechanisms underlying how the information of early development is epigenetically imprinted in OPCs in adults. Our study sheds light on OPC‐based cell therapy using OPC transplantation. Our study suggests that the origin of OPCs must be considered to ensure efficient therapeutic outcomes. We also provide an approach to reprogram ventral OPCs and avoid their death after transplantation by manipulating the *NF1* gene, although more research is warranted to fully decipher the underlying mechanism. In terms of cancer biology, although ventrally derived OPCs diminished at an older age during normal development, they still could break this limit and function as a cell origin of brain tumors after acquiring certain oncogenic mutations, such as *NF1* deficiency.

## 4 Experimental Section

### Plasmids Construction—hyperPBase

The hyperPBase fragment were cloned into the vector named Piggy‐GreenShirt between the EcoRV and SalI site by standard restriction digestion procedures.

### Plasmids Construction—PB Reporter Vector

The plasmid containing the LoxP‐ nΦYFP‐Stop‐Loxp‐EGFP/tdTomato cassette was constructed on the PSK^+^ vector using In‐Fusion cloning kit (Takara). The LoxP‐ nΦYFP‐Stop‐Loxp‐EGFP/tdTomato cassette was then flanked by two insulator sequences and inverted terminal repeat (ITR) sequence of PiggyBac transposon on each side for ensuring stable expression and recognition of the hyperPBase.

### Plasmids Construction—CMV‐FlpO

The FlpO fragment were cloned into the vector named Piggy‐GreenShirt between the EcoRV and SalI site by standard restriction digestion procedures.

### Plasmids Construction—PB‐Cas9 Vector

The Cas9‐P2A‐FlpO fragment was amplified by overlapping PCR and replaced the EGFP sequence on the PB reporter vector. Then the sgRNAs of *NF1* or NT were ligated tandemly through Gold Gate reaction and cloned into the between the MfeI and SpeI site by standard restriction digestion procedures.

The sequences of sgRNAs are listed below:

**Table:** 

sgRNA	Sequence
Sg‐*NF1*‐1	AGTCAGCACCGAGCACAACA
Sg‐*NF1*‐2	GACCAGAGAAAACTTGTATT
Sg‐*NF1*‐3	GGTGGAATGGGTCCAGGCCG
Sg‐NT	GCGACCAATACGCGAACGTC

### Mouse Lines

All animal procedures were accordance with the animal care guidelines approved by the Institutional Animal Care and Use Committee of Zhejiang University School of Medicine. The female ICR mice were purchased from Shanghai Slac laboratory animal CO.LTD. *NG2‐Cre* (Stock No. 0 08533, JAX), *NG2‐Cre^ERT^* (Stock No. 0 08533, JAX), and *hGFAP‐Cre* (Stock No. 0 04600, JAX) were bred with female ICR mice to generate heterozygous embryos for IUE. The day of vaginal plug was considered as the embryonic day (E)0.5. IUE was performed on the embryos of around E14.5. *TG11ML* (Stock No.022977, JAX), *GT11ML* (Stock No.022976, JAX) and *NG2‐Cre^ERT^* (Stock No. 0 08533, JAX) were crossed together to generate MADM models. *Rosa26CAG‐FSF‐LSL‐tdTomao (or Ai65(RCFL‐tdT)‐D)* (Stock No.021875, JAX) and *NG2‐Cre^ERT^* (Stock No. 0 08533, JAX) were crossed together to generate *Ai65(RCFL‐tdT)‐D; NG2‐Cre^ERT^* which were bred with ICR later for IUE.

### In Utero Electroporation

The operation of IUE was performed according to the previous studies^[^ ^69^, ^70^, ^71^ ^]^ by using the ECM830 system and 5mm tweezer trodes (CUY650P5). HyperPBase and PB‐LSL‐EGFP/tdTomato plasmids were mixed with fast green (finally 0.1%) and injected into the ventricle of E14.5 embryos. In order to being most feasible and effective, a series of tests were carried out and the parameters of IUE were finally optimized as: the voltage of 50V, lasting 100 ms, separated by 900 ms, and a chain of 5‐pulses in total.

### Tamoxifen and BrdU Administration

For in vivo clonal analysis of *NG2‐Cre^ERT^* model, the P3 pups after IUE were injected with 25 µg Tamoxifen subcutaneously under their back skin. Tamoxifen (Sigma) was dissolved in the autoclaved corn oil at 0.5mg mL^−1^. In the titration experiment of the dosage of tamoxifen as shown in Figure 3, the concentration of 1mg mL^−1^ and more times of injection were also administrated. Same volume of corn oil vehicle was injected as the background control.

For analysis of *Ai65(RCFL‐tdT)‐D; NG2‐Cre^ERT^* model, P3 pups after IUE were injected with 1mg Tamoxifen subcutaneously under their back skin. Tamoxifen (Sigma) was dissolved in the autoclaved corn oil at 20 mg mL^−1^.

For BrdU labeling of OPC clones, P3 pups after IUE were injected with 0.2mg BrdU subcutaneously under their back skin at following the Tamoxifen administration. BrdU (Sigma) was dissolved in the autoclaved PBS at 5mg mL^−1^. For OPC populations, BrdU was administrated at 62.5mg kg^−1^ body weight intraperitoneally at P53 for 8 days (one dose a day). BrdU (Sigma) was dissolved in the autoclaved PBS at 5mg mL^−1^.

### Tissue Procession and Immunofluorescence

Mice were anesthetized and transcardially perfused with cold PBS, followed by 4% paraformaldehyde. After dissected, the brains were post‐fixed in 4% PFA for 24 h and then washed 3× in PBS before dehydrated in 30% sucrose. After 48 h in sucrose, the brains were embedded in the optimal cutting temperature (O.C.T.) compound and snap‐frozen on the dry ice, then stored at −80 °C.

Brain tissues were cryo‐sectioned consecutively into 20 µm thick slices. The brain sections were dried at RT and 60 °C for an hour separately. After that, the sections were post‐fixed with 2% PFA for 15 min, then washed in PBS three times for 10 min each time. Rinsing with PBT (0.3% Triton X‐100 in PBS) for 1 min and incubating with blocking solution (5% normal donkey serum in PBT) for 20 min were performed to avoid nonspecific binding of antibodies. The slices were incubated overnight with primary antibody at 4 °C. The secondary antibodies incubation was implemented overnight at 4 °C, following washing in PBT four times for 10 min each time. Last, the slides were washed in the same way and incubated in DAPI solution (25 ng mL^−1^ in PBS) for 20 min. After washing in PBS for 5 min, the slides were mounted with 65% glycerol in PBS. The antibodies are listed below:

**Table:** 

Antigen	Species	Dilution	Source	Catalog no.
GFP	Chicken	1:1000	Aves	GFP‐1020
PDGFR*α*	Goat	1:200	R&D	AF1062
APC‐CC1	Mouse	1:50	Millipore	OP80
phiYFP	Rabbit	1:10000	Evrogen	AB601
PDGFR*β*	Goat	1:200	R&D	AF1042
GFAP	Mouse	1:800	Millipore	MAB360
BrdU	Rat	1:125	Abcam	ab6326
Ki67	Rabbit	1:500	Abcam	ab15580
MBP	Mouse	1:1000	Biolegend	808 402
Caspr	Rabbit	1:800	CST	97736S
NF‐H	Rabbit	1:100	Abcam	ab207176
DsRed	Goat	1:200	Santa Cruz	Sc‐33353
*β*III‐tubulin	Mouse	1:3000	Abcam	ab78078
Pdgfr*α*	Rat	1:200	eBioscience	14‐1401‐82
cMyc	Goat	1:400	Novas	NB600‐338

### Collection of Clones

The consecutive sections were scanned serially using the multi‐purpose zoom microscope (Nikon AZ100) to record the positions of clones, which were defined as a cluster of GFP‐labeled cells derived from the common one progenitor and quite far away from other GFP‐labeled cells. The magnified images were acquired via the laser scanning confocal microscope (Olympus FV3000).

The subtypes of clones were defined according to their orientations. The clones containing <5 cells could not be molded into any shapes. The clone, whose longest diameter were less than or equal to 1.5× the shortest diameter, was regarded as “sphere‐like” subtype. Otherwise, if the longest diameter of a clone was parallel to CC, this clone would be classified as “horizontal” subtype. Or if perpendicular to CC, this clone was seen as “radial” subtype. In case the orientation of the longest diameter of a clone was from rostral to caudal, it would be referred to as “R/C” subtype.

### 3D Reconstruction and Computational Modeling

The images of serial coronal sections were collected via the multi‐purpose zoom microscope (Nikon AZ100) in sequence from rostral to caudal, as the formwork provided to Neurolucida (MBF) for reconstructing. All labeled cells were marked in Neurolucida and the coordinates of each labeled cells in the brain were determined. The stereotaxic brain atlas^[^ ^37^ ^]^ was used as the standard to locate the brain anatomical structures where labeled cells were positioned.

### NND Analysis

The NND analysis were performed through the home‐made scripts in Matlab (Mathworks). The distribution of NND of the dataset could reveal their spatial point pattern in the specific area. According to the previous studies,^[^ ^39^, ^41^, ^44^ ^]^ The codes were based on the Matlab documentation of knnsearch. Equation (1): finds the nearest neighbor and distance in spatial **Data1** for each query point in spatial **Data2** and returns the indices of the nearest neighbors in **Idx** and **Dist**. Equation (2): for each cell i the distance to its closest neighbor was measured and denoted as **Dist**_i_, **num** was used to record the cells number, which the cells **Dist**_i_ ≤ *y*. where *y* denotes the gradient matrix of distance, and the ranges was from 1 to the max distance in the **Data1** spatial. Thus, the cumulative distribution function of NND is **G(y)** (Equation (3)).

(1) $$f\left(\mathrm{Idx},\mathrm{Dist}\right)=\mathrm{knnsearch}\left(\mathrm{Data}1,\mathrm{Data}2\right)$$

(2) $$F\left(\mathrm{num}\left(i\right),\;i\right)=\left\{\begin{array}{c} 1,\;if\;Dist\;\leq \;y\left(i\right)\\ 0,\;\mathrm{else}\; \end{array}\right.$$

(3) $$G\left(y\right)\;=\sum_{i=1}^{N}F(\mathrm{num}(i),\;i)\;/N$$

A dataset of random points was simulated 100× in the same space volume and same number as the data from brain samples. If the cumulative distance of cells is shorter than the random one, the cells were clustered. The dispersed cells have longer distance.

### Statistical Analysis

Statistical analysis was carried out using GraphPad Prism 8 (GraphPad Software), and Matlab (Mathworks). Continuous variables are expressed as means ± SEM. Data were analyzed first by Shapiro–Wilk test to check their normality. For normally distributed data sets, unpaired t test was used to compare two groups and one‐way ANOVA followed by a Tukey post‐hoc test was carried out across groups. For non‐normally distributed data sets, nonparametric Mann‐Whitney or Kruskal‐Wallis test was carried out. Two‐way ANOVA test followed by Tukey post‐hoc test was applied to analyze data sets with two variables. The specific statistical approach is described in the legend of each figure. In all case, significance was defined as *P* ≤ 0.05.

## Conflict of Interest

The authors declare no conflict of interest.

## Author Contributions

C.L. conceptualized the project. R.L. and C.L. initiated this study. C.L. supervised the study and conducted quality control on the data. C.L. and R.L. wrote the manuscript with inputs from all authors. R.L. performed most experiments and produced all figures. Y.J. and R.B edited the codes of mathematical models. P.G. performed part of immunofluorescent experiments. W.J. performed the RNA‐seq experiments.

## Supporting information

Supporting Information

Supplemental Video 1

Supplemental Video 2

Supplemental Video 3

Supplemental Video 4

## Data Availability

The data that support the findings of this study are openly available in GENE EXPRESSION OMNIBUS at https://doi.org/10.1002/advs.202001724, reference number GSE108862.

## References

[1] A. Nishiyama , R. Suzuki , X. Zhu , Front. Neurosci. 2014, 8, 133. 25018689 10.3389/fnins.2014.00133 PMC4072963

[2] L. Dimou , C. Simon , F. Kirchhoff , H. Takebayashi , M. Gotz , J. Neurosci. 2008, 28, 10434. 18842903 10.1523/JNEUROSCI.2831-08.2008 PMC6671038

[3] S. Geha , J. Pallud , M. P. Junier , B. Devaux , N. Leonard , F. Chassoux , H. Chneiweiss , C. Daumas‐Duport , P. Varlet , Brain Pathol. 2010, 20, 399. 19486010 10.1111/j.1750-3639.2009.00295.x PMC8094800

[4] T. Kondo , M. Raff , Science 2000, 289, 1754. 10976069 10.1126/science.289.5485.1754

[5] D. E. Bergles , J. D. B. Roberts , P. Somogyi , C. E. Jahr , Nature 2000, 405, 187. 10821275 10.1038/35012083

[6] J. L. Ziskin , A. Nishiyama , M. Rubio , M. Fukaya , D. E. Bergles , Nat. Neurosci. 2007, 10, 321. 17293857 10.1038/nn1854 PMC2140234

[7] I. A. McKenzie , D. Ohayon , H. Li , J. P. de Faria , B. Emery , K. Tohyama , W. D. Richardson , Science 2014, 346, 318. 25324381 10.1126/science.1254960 PMC6324726

[8] S. Marques , A. Zeisel , S. Codeluppi , D. van Bruggen , A. Mendanha Falcao , L. Xiao , H. Li , M. Haring , H. Hochgerner , R. A. Romanov , D. Gyllborg , A. B. Munoz‐Manchado , G. La Manno , P. Lonnerberg , E. M. Floriddia , F. Rezayee , P. Ernfors , E. Arenas , J. Hjerling‐Leffler , T. Harkany , W. D. Richardson , S. Linnarsson , G. Castelo‐Branco , Science 2016, 352, 1326. 27284195 10.1126/science.aaf6463 PMC5221728

[9] T. Kuhlmann , V. Miron , Q. Cui , C. Wegner , J. Antel , W. Bruck , Brain 2008, 131, 1749. 18515322 10.1093/brain/awn096

[10] M. Graciarena , A. Seiffe , B. Nait‐Oumesmar , A. M. Depino , Front. Cell Neurosci. 2018, 12, 517. 30687009 10.3389/fncel.2018.00517 PMC6338056

[11] J. Fitzgerald , L. Gallagher , J. McGrath , J. Autism Dev. Disord. 2019, 49, 2664. 27207090 10.1007/s10803-016-2803-8

[12] N. Takahashi , T. Sakurai , K. L. Davis , J. D. Buxbaum , Prog. Neurobiol. 2011, 93, 13. 20950668 10.1016/j.pneurobio.2010.09.004 PMC3622281

[13] D. R. Dries , Y. Zhu , M. M. Brooks , D. A. Forero , M. Adachi , B. Cenik , J. M. West , Y. H. Han , C. Yu , J. Arbella , A. Nordin , R. Adolfsson , J. Del‐Favero , Q. R. Lu , P. Callaerts , S. G. Birnbaum , G. Yu , J. Biol. Chem. 2016, 291, 11647. 27008863 10.1074/jbc.M116.715078 PMC4882434

[14] A. I. Persson , C. Petritsch , F. J. Swartling , M. Itsara , F. J. Sim , R. Auvergne , D. D. Goldenberg , S. R. Vandenberg , K. N. Nguyen , S. Yakovenko , J. Ayers‐Ringler , A. Nishiyama , W. B. Stallcup , M. S. Berger , G. Bergers , T. R. McKnight , S. A. Goldman , W. A. Weiss , Cancer Cell 2010, 18, 669. 21156288 10.1016/j.ccr.2010.10.033 PMC3031116

[15] C. Liu , J. C. Sage , M. R. Miller , R. G. W. Verhaak , S. Hippenmeyer , H. Vogel , O. Foreman , R. T. Bronson , A. Nishiyama , L. Luo , H. Zong , Cell 2011, 146, 209. 21737130 10.1016/j.cell.2011.06.014 PMC3143261

[16] M. Monje , S. S. Mitra , M. E. Freret , T. B. Raveh , J. Kim , M. Masek , J. L. Attema , G. Li , T. Haddix , M. S. B. Edwards , P. G. Fisher , I. L. Weissman , D. H. Rowitch , H. Vogel , A. J. Wong , P. A. Beachy , Proc. Natl. Acad. Sci. U. S. A. 2011, 108, 4453. 21368213 10.1073/pnas.1101657108 PMC3060250

[17] L. Zhang , X. He , X. Liu , F. Zhang , L. F. Huang , A. S. Potter , L. Xu , W. Zhou , T. Zheng , Z. Luo , K. P. Berry , A. Pribnow , S. M. Smith , C. Fuller , B. V. Jones , M. Fouladi , R. Drissi , Z.‐J. Yang , W. C. Gustafson , M. Remke , S. L. Pomeroy , E. J. Girard , J. M. Olson , A. S. Morrissy , M. C. Vladoiu , J. Zhang , W. Tian , M. Xin , M. D. Taylor , S. S. Potter , M. F. Roussel , W. A. Weiss , Q. R. Lu , Cancer Cell 2019, 36, 302. 31474569 10.1016/j.ccell.2019.07.009 PMC6760242

[18] F. Viganò , L. Dimou , Brain Res. 2016, 1638, 129. 26388262 10.1016/j.brainres.2015.09.012

[19] J. Trotter , K. Karram , A. Nishiyama , Brain Res. Rev. 2010, 63, 72. 20043946 10.1016/j.brainresrev.2009.12.006 PMC2862831

[20] C. Bjartmar , C. Hildebrand , K. Loinder , Glia 1994, 11, 235. 7960028 10.1002/glia.440110304

[21] M. Dawson , Mol. Cell. Neurosci. 2003, 24, 476. 14572468 10.1016/s1044-7431(03)00210-0

[22] K. Psachoulia , F. Jamen , K. M. Young , W. D. Richardson , Neuron. Glia Biol. 2009, 5, 57. 20346197 10.1017/S1740925X09990354 PMC6329448

[23] X. Zhu , R. A. Hill , D. Dietrich , M. Komitova , R. Suzuki , A. Nishiyama , Development 2011, 138, 745. 21266410 10.1242/dev.047951 PMC3026417

[24] R. A. Hill , K. D. Patel , J. Medved , A. M. Reiss , A. Nishiyama , J. Neurosci. 2013, 33, 14558. 24005306 10.1523/JNEUROSCI.2001-12.2013 PMC3761056

[25] S. Marques , D. van Bruggen , D. P. Vanichkina , E. M. Floriddia , H. Munguba , L. Väremo , S. Giacomello , A. M. Falcão , M. Meijer , Å. K. Björklund , J. Hjerling‐Leffler , R. J. Taft , G. Castelo‐Branco , Dev. Cell 2018, 46, 504. 30078729 10.1016/j.devcel.2018.07.005 PMC6104814

[26] J. E. Davies , R. H. Miller , Dev. Biol. 2001, 233, 513. 11336511 10.1006/dbio.2001.0224

[27] E. M. Perez Villegas , C. Olivier , N. Spassky , C. Poncet , P. Cochard , B. Zalc , J. L. Thomas , S. Martinez , Dev. Biol. 1999, 216, 98. 10588866 10.1006/dbio.1999.9438

[28] E. Noll , R. H. Miller , Development 1993, 118, 563. 8223279 10.1242/dev.118.2.563

[29] A. Vallstedt , J. M. Klos , J. Ericson , Neuron 2005, 45, 55. 15629702 10.1016/j.neuron.2004.12.026

[30] M. Fogarty , W. D. Richardson , N. Kessaris , Development 2005, 132, 1951. 15790969 10.1242/dev.01777

[31] N. Kessaris , M. Fogarty , P. Iannarelli , M. Grist , M. Wegner , W. D. Richardson , Nat. Neurosci. 2006, 9, 173. 16388308 10.1038/nn1620 PMC6328015

[32] J. Cai , Y. Qi , X. Hu , M. Tan , Z. Liu , J. Zhang , Q. Li , M. Sander , M. Qiu , Neuron 2005, 45, 41. 15629701 10.1016/j.neuron.2004.12.028

[33] L. Pinto , M. Gotz , Prog. Neurobiol. 2007, 83, 2. 17580100 10.1016/j.pneurobio.2007.02.010

[34] L. Zhuo , M. Theis , I. Alvarez‐Maya , M. Brenner , K. Willecke , A. Messing , Genesis 2001, 31, 85. 11668683 10.1002/gene.10008

[35] X. Zhu , R. A. Hill , A. Nishiyama , Neuron. Glia Biol. 2008, 4, 19. 19006598 10.1017/S1740925X09000015

[36] X. Zhu , D. E. Bergles , A. Nishiyama , Development 2008, 135, 145. 18045844 10.1242/dev.004895

[37] K. Franklin , G. Paxinos , The Mouse Brain in Stereotaxic Coordinates, Academic Press, San Diego, CA 1997.

[38] M. A. Bonaguidi , M. A. Wheeler , J. S. Shapiro , R. P. Stadel , G. J. Sun , G. L. Ming , H. Song , Cell 2011, 145, 1142. 21664664 10.1016/j.cell.2011.05.024 PMC3124562

[39] P. Gao , M. P. Postiglione , T. G. Krieger , L. Hernandez , C. Wang , Z. Han , C. Streicher , E. Papusheva , R. Insolera , K. Chugh , O. Kodish , K. Huang , B. D. Simons , L. Luo , S. Hippenmeyer , S. H. Shi , Cell 2014, 159, 775. 25417155 10.1016/j.cell.2014.10.027 PMC4225456

[40] J. Garcia‐Marques , R. Nunez‐Llaves , L. Lopez‐Mascaraque , J. Neurosci. 2014, 34, 2305. 24501369 10.1523/JNEUROSCI.3060-13.2014 PMC6608533

[41] K. N. Brown , S. Chen , Z. Han , C. H. Lu , X. Tan , X. J. Zhang , L. Ding , A. Lopez‐Cruz , D. Saur , S. A. Anderson , K. Huang , S. H. Shi , Science 2011, 334, 480. 22034427 10.1126/science.1208884 PMC3304494

[42] G. Ciceri , N. Dehorter , I. Sols , Z. J. Huang , M. Maravall , O. Marin , Nat. Neurosci. 2013, 16, 1199. 23933753 10.1038/nn.3485

[43] C. C. Harwell , L. C. Fuentealba , A. Gonzalez‐Cerrillo , P. R. Parker , C. C. Gertz , E. Mazzola , M. T. Garcia , A. Alvarez‐Buylla , C. L. Cepko , A. R. Kriegstein , Neuron 2015, 87, 999. 26299474 10.1016/j.neuron.2015.07.030 PMC4581718

[44] H. T. Xu , Z. Han , P. Gao , S. He , Z. Li , W. Shi , O. Kodish , W. Shao , K. N. Brown , K. Huang , S. H. Shi , Cell 2014, 157, 1552. 24949968 10.1016/j.cell.2014.03.067 PMC4120073

[45] L. Madisen , A. R. Garner , D. Shimaoka , A. S. Chuong , N. C. Klapoetke , L. Li , A. van der Bourg , Y. Niino , L. Egolf , C. Monetti , H. Gu , M. Mills , A. Cheng , B. Tasic , T. N. Nguyen , S. M. Sunkin , A. Benucci , A. Nagy , A. Miyawaki , F. Helmchen , R. M. Empson , T. Knopfel , E. S. Boyden , R. C. Reid , M. Carandini , H. Zeng , Neuron 2015, 85, 942. 25741722 10.1016/j.neuron.2015.02.022 PMC4365051

[46] H. Zong , J. S. Espinosa , H. H. Su , M. D. Muzumdar , L. Luo , Cell 2005, 121, 479. 15882628 10.1016/j.cell.2005.02.012

[47] S. Hippenmeyer , Y. H. Youn , H. M. Moon , K. Miyamichi , H. Zong , A. Wynshaw‐Boris , L. Luo , Neuron 2010, 68, 695. 21092859 10.1016/j.neuron.2010.09.027 PMC3044607

[48] M. D. Muzumdar , L. Luo , H. Zong , Proc. Natl. Acad. Sci. U. S. A. 2007, 104, 4495. 17360552 10.1073/pnas.0606491104 PMC1810340

[49] J. S. Espinosa , D. G. Wheeler , R. W. Tsien , L. Luo , Neuron 2009, 62, 205. 19409266 10.1016/j.neuron.2009.03.006 PMC2788338

[50] R. Beattie , M. P. Postiglione , L. E. Burnett , S. Laukoter , C. Streicher , F. M. Pauler , G. Xiao , O. Klezovitch , V. Vasioukhin , T. H. Ghashghaei , S. Hippenmeyer , Neuron 2017, 94, 517. 28472654 10.1016/j.neuron.2017.04.012

[51] A. Tian , B. Kang , B. Li , B. Qiu , W. Jiang , F. Shao , Q. Gao , R. Liu , C. Cai , R. Jing , W. Wang , P. Chen , Q. Liang , L. Bao , J. Man , Y. Wang , Y. Shi , J. Li , M. Yang , L. Wang , J. Zhang , S. Hippenmeyer , J. Zhu , X. Bian , Y.‐J. Wang , C. Liu , Adv. Sci. 2020, 7, 2001724. 10.1002/advs.202001724 PMC7610337 33173731

[52] S. H. Kang , Y. Li , M. Fukaya , I. Lorenzini , D. W. Cleveland , L. W. Ostrow , J. D. Rothstein , D. E. Bergles , Nat. Neurosci. 2013, 16, 571. 23542689 10.1038/nn.3357 PMC3637847

[53] D. M. Walsh , P. T. Roth , W. R. Holmes , K. A. Landman , T. D. Merson , B. D. Hughes , J. Theor. Biol. 2016, 406, 17. 27343034 10.1016/j.jtbi.2016.06.028

[54] J. S. Lee , A. Padmanabhan , J. Shin , S. Zhu , F. Guo , J. P. Kanki , J. A. Epstein , A. T. Look , Hum. Mol. Genet. 2010, 19, 4643. 20858602 10.1093/hmg/ddq395 PMC3999377

[55] C. Liu , J. C. Sage , M. R. Miller , R. G. Verhaak , S. Hippenmeyer , H. Vogel , O. Foreman , R. T. Bronson , A. Nishiyama , L. Luo , H. Zong , Cell 2011, 146, 209. 21737130 10.1016/j.cell.2011.06.014 PMC3143261

[56] P. P. Gonzalez , J. Kim , R. P. Galvao , N. Cruickshanks , R. Abounader , H. Zong , Glia 2018, 66, 999. 29392777 10.1002/glia.23297 PMC7808243

[57] A. Tian , B. Kang , B. Li , B. Qiu , W. Jiang , F. Shao , Q. Gao , R. Liu , C. Cai , R. Jing , W. Wang , P. Chen , Q. Liang , L. Bao , J. Man , Y. Wang , Y. Shi , J. Li , M. Yang , L. Wang , J. Zhang , S. Hippenmeyer , J. Zhu , X. Bian , Y. J. Wang , C. Liu , Adv. Sci. 2020, 7, 2001724. 10.1002/advs.202001724 PMC7610337 33173731

[58] M. C. Raff , L. E. Lillien , W. D. Richardson , J. F. Burne , M. D. Noble , Nature 1988, 333, 562. 3287177 10.1038/333562a0

[59] B. A. Barres , I. K. Hart , H. S. Coles , J. F. Burne , J. T. Voyvodic , W. D. Richardson , M. C. Raff , Cell 1992, 70, 31. 1623522 10.1016/0092-8674(92)90531-g

[60] F. B. Gao , M. Raff , J. Cell Biol. 1997, 138, 1367. 9298991 10.1083/jcb.138.6.1367 PMC2132550

[61] D. G. Tang , Y. M. Tokumoto , M. C. Raff , J. Cell Biol. 2000, 148, 971. 10704447 10.1083/jcb.148.5.971 PMC2174541

[62] X. Zhu , H. Zuo , B. J. Maher , D. R. Serwanski , J. J. LoTurco , Q. R. Lu , A. Nishiyama , Development 2012, 139, 2299. 22627280 10.1242/dev.078873 PMC3367441

[63] Y. Zhang , K. Chen , S. A. Sloan , M. L. Bennett , A. R. Scholze , S. O'Keeffe , H. P. Phatnani , P. Guarnieri , C. Caneda , N. Ruderisch , S. Deng , S. A. Liddelow , C. Zhang , R. Daneman , T. Maniatis , B. A. Barres , J. Q. Wu , J. Neurosci. 2014, 34, 11929. 25186741 10.1523/JNEUROSCI.1860-14.2014 PMC4152602

[64] S. Temple , M. C. Raff , Cell 1986, 44, 773. 3948247 10.1016/0092-8674(86)90843-3

[65] B. A. Barres , M. A. Lazar , M. C. Raff , Development 1994, 120, 1097. 8026323 10.1242/dev.120.5.1097

[66] F. B. Gao , B. Durand , M. Raff , Curr. Biol. 1997, 7, 152. 9016704 10.1016/s0960-9822(06)00060-1

[67] J. C. Dugas , A. Ibrahim , B. A. Barres , J. Neurosci. 2007, 27, 6185. 17553990 10.1523/JNEUROSCI.0628-07.2007 PMC6672145

[68] H. Wichterle , D. H. Turnbull , S. Nery , G. Fishell , A. Alvarez‐Buylla , Development 2001, 128, 3759. 11585802 10.1242/dev.128.19.3759

[69] E. Pacary , F. Guillemot , Methods Mol. Biol. 2014, 1082, 285. 24048941 10.1007/978-1-62703-655-9_19

[70] T. Saito , N. Nakatsuji , Dev. Biol. 2001, 240, 237. 11784059 10.1006/dbio.2001.0439

[71] H. Tabata , K. Nakajima , Neuroscience 2001, 103, 865. 11301197 10.1016/s0306-4522(01)00016-1

