# Supplementary material for: In Vivo Clonal Analysis Reveals Development Heterogeneity of Oligodendrocyte Precursor Cells Derived from Distinct Germinal Zones
Source: Adv Sci (Weinh). 2021 Aug 16;8(20):2102274. doi: 10.1002/advs.202102274 (PMC8529438; doi:10.1002/advs.202102274)
Supplement: Supplementary file 1 — Supporting Information [file ADVS-8-2102274-s001.pdf]

© 2021 Wiley-VCH GmbH

ADVANCED SCIENCE
Open Access

# Supporting Information

for *Adv. Sci.,* DOI: 10.1002/advs.202102274

# In Vivo Clonal Analysis Reveals Development Heterogeneity of Oligodendrocyte Precursor Cells Derived from Distinct Germinal Zones

*Rui Liu, Yinhang Jia, Peng Guo, Wenhong Jiang, Ruiliang Bai and Chong Liu**

Supporting Information

# In vivo clonal analysis reveals development heterogeneity of oligodendrocyte precursor cells derived from distinct germinal zones

*Rui Liu, Yinhang Jia, Ruiliang Bai, Peng Guo, Wenhong Jiang and Chong Liu**

## Figure S1.

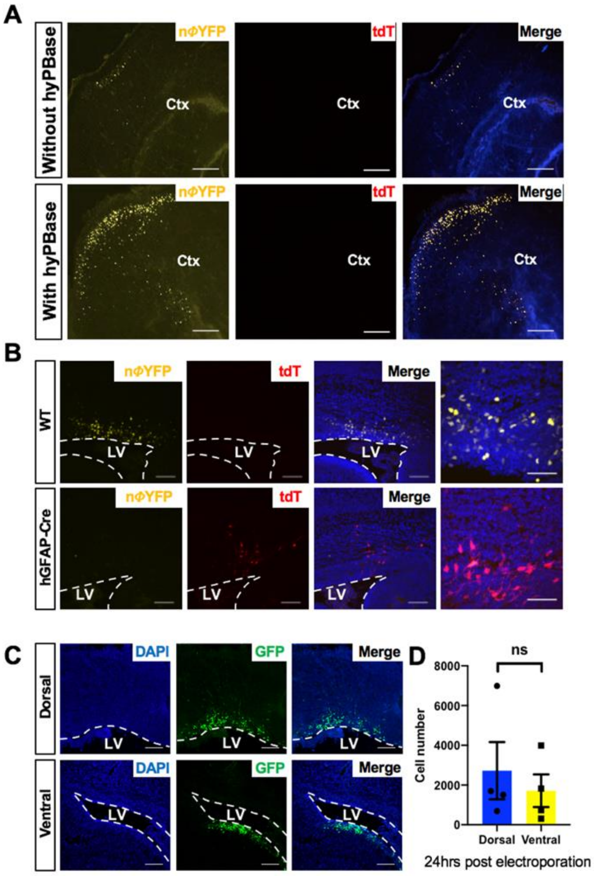


**Figure S1**. **Validation of the PiggyBac transposon-based in vivo lineage tracing system.**

**(A)** HyperPBase is critical for the stable integration of the PB vectors into the embryonic neural stem cells. Ctx, cortex. Scale bars,200μm.

**(B)** Validation of the Cre-dependent expression of reporters in the system. tdTomato can only be detected in the mouse line expressing *hGFAP-Cre*. The boundary of the lateral ventricle wall is demarcated by dot lines. LV, Lateral ventricle. The right panels are the magnifications of the third panels. Scale bars: low magnificent, 80μm, higher magnificent, 50μm.

**(C)** Representative immunofluorescent images of GFP+ cells along the lateral ventricle at 24hrs post-electroporation. Scale bars: 100μm.

**(D)** Quantification of the initially labeling efficiency of the electroporation. Error bars: mean ± SEM. N=4 for each group. One-tailed T test. ns, not significant.

**Figure S2.**

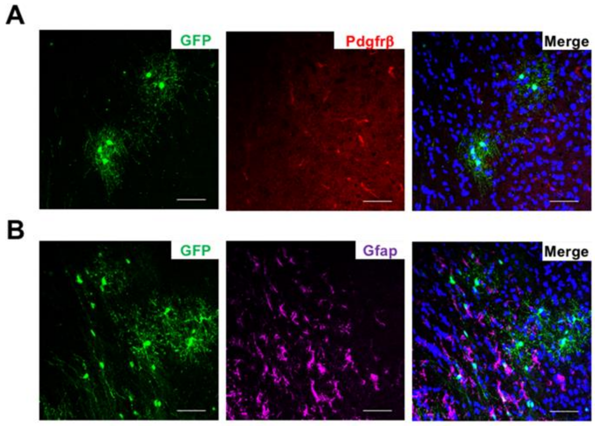


**Figure S2. Pericytes and astrocytes were not be labeled in the *NG2-Cre* electroporated model.**

Representative immunofluorescent images of labeled oligodendrocyte lineage cells stained with **(A)** Pdgfrβ, a pericyte marker, and **(B)** Gfap, an astrocyte marker. Scale bars, 50μm.

## Figure S3.

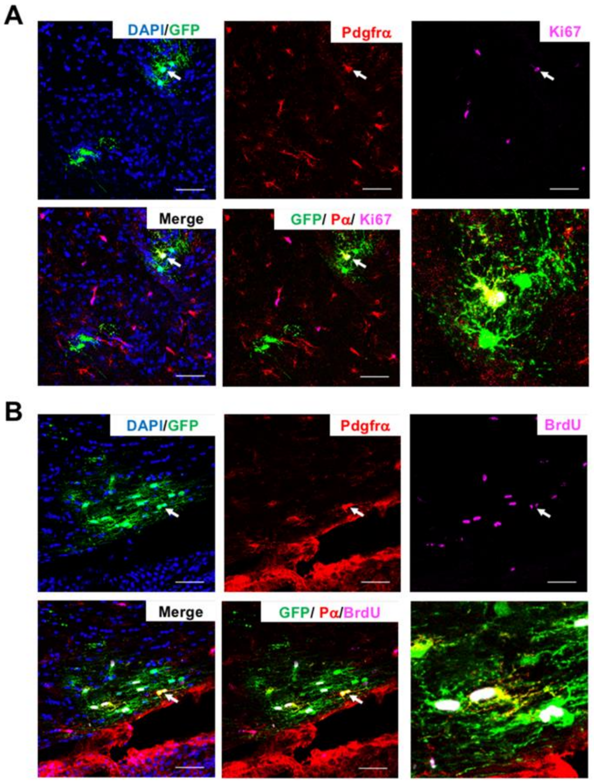


**Figure S3**. **Representative staining images of dividing EGFP positive OPCs.**

**(A)** Ki67 and **(B)** BrdU immunofluorescence was performed to indicate the dividing or divided cells at specific times. Scale bars, 50μm. The cells indicated by arrows are shown in the zoomed-in images.

# Figure S4.

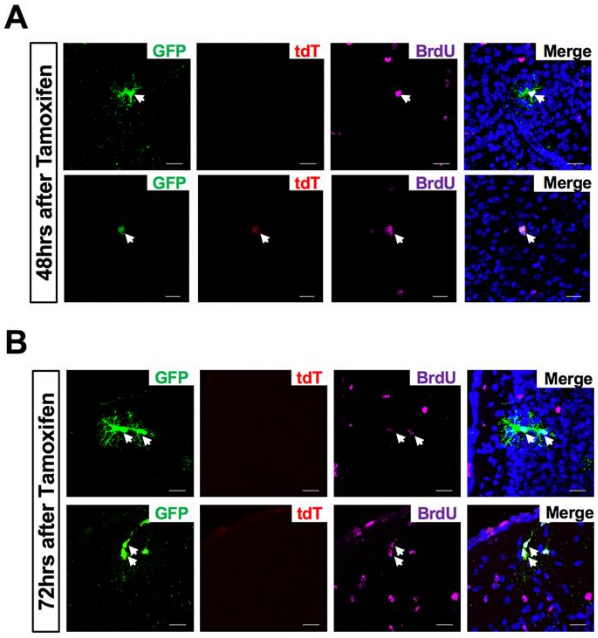


**Figure S4**. **Administration of BrdU to prove the clonality of OPC clusters.**

**(A)** Representative confocal images of single cells labeled with BrdU at 48 hours post injection. Scale bars, 20µm.

**(B)** Representative confocal images of twin-spots labeled with BrdU at 48 hours post injection. Scale bars, 20µm.

# Figure S5.

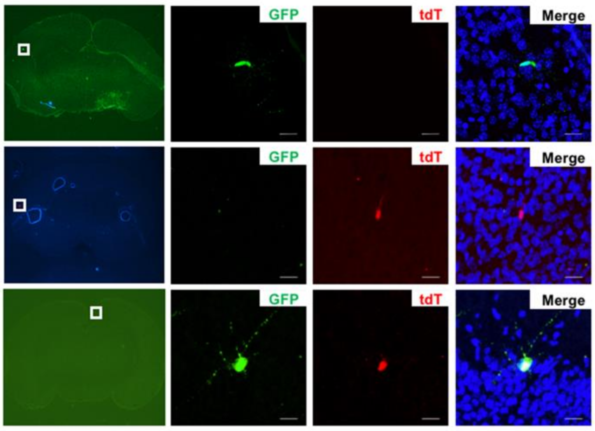


**Figure S5. Representative confocal images of green (EGFP$^+$), red (tdTomato$^+$) and yellow (EGFP$^+$tdTomato$^+$) cells generated in the system for NND analysis.**

## Figure S6.

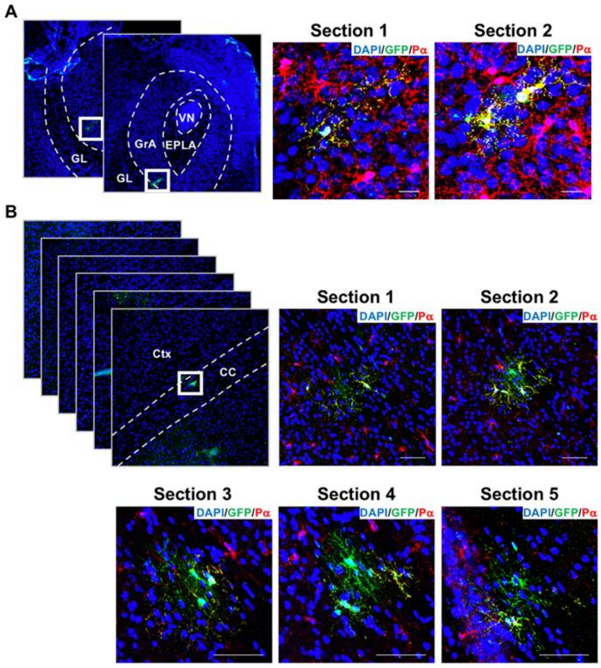


**Figure S6**. **Some OPC clones occupy unique brain areas.**

**(A)** Representative images of a clone existing in the olfactory bulb. VN, vomeronasal nerve. EPLA, external plexiform layer of the accessory olfactory bulb. GrA, granule cell layer of the accessory olfactory bulb. GL, glomerular layer of the olfactory bulb. Scale bars, 20µm.

**(B)** Representative images of a clone occupying both the CC and the cortex. Ctx, cortex. CC, corpus callosum. Scale bars, 50µm.

**Figure S7.**

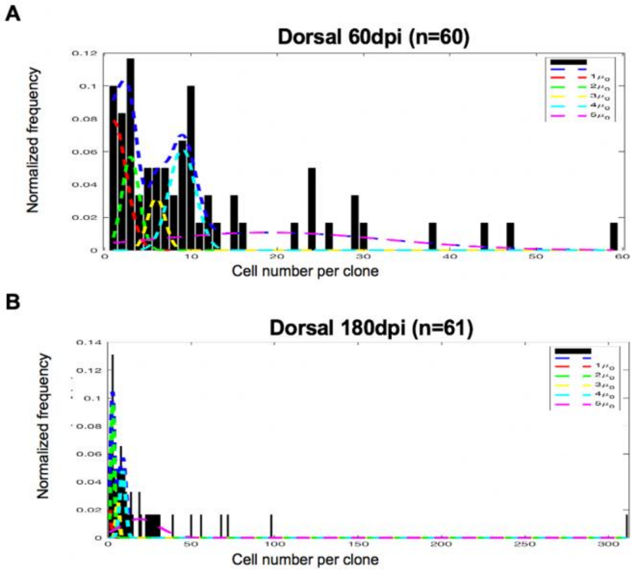


**Figure S7**. Gaussian Fitting of frequency distribution of the sizes of dorsally derived clones at A) 60dpi, B)180dpi. N number was indicated above the graph.

# Figure S8.

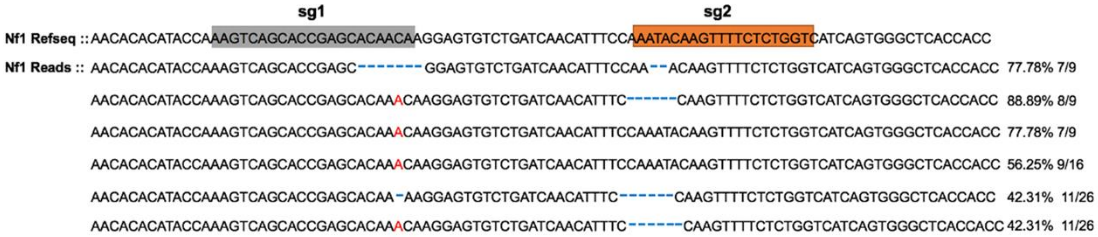


**Figure S8**. **The RNA-seq of targeted NF1 genome sequence sites edited by sgRNA against NF1.**

The RNA sequencing results proved successful editing at the target sites desired in this study. The first line shows the wild-type sequence of NF1. The lines below show the sequencing reads of the samples. The proportion of mutations is shown on the right.

**Figure S9.**

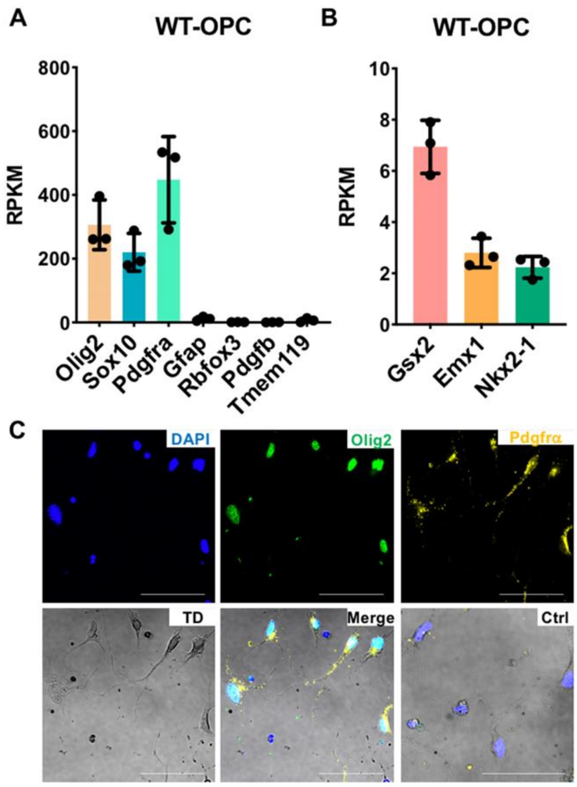


**Figure S9**. **The transcriptome data reveal that OPCs express Gsx2.**

**(A)** RNA-seq data validates that the enriched OPCs strongly expressed OPC lineage marker Olig2, Sox10, and Pdgfrα and hardly expressed Gfap, Rbfox3 (also named as NeuN), Pdgfrβ, and Tmem119. Wild-type OPCs were enriched from the P8 ICR mouse forebrain by the immunopanning approach.

**(B)** RNA-seq data showed the expression of Gsx2 in OPCs enriched in (A).

**(C)** Representative images of enriched OPCs used for the RNA sequencing.

**Video S1**. Representative video of a clone classified as “Horizontal” subtype related to figure 6. OPCs were marked as red spheres, oligodendrocytes as cyan dots.

**Video S2**. Representative video of a clone classified as “Radial” subtype related to figure 6. OPCs were marked as red spheres, oligodendrocytes as cyan dots.

**Video S3**. Representative video of a clone classified as “Rostral-Caudal” subtype related to figure 6. OPCs were marked as red spheres, oligodendrocytes as cyan dots.

**Video S4**. Representative video of a clone classified as “Sphere-like” subtype related to figure 6. OPCs were marked as red spheres, oligodendrocytes as cyan dots.
